# Supplementary material for: NeuroD1 gene therapy inhibits glioma growth and extends life span through in vivo reprogramming approach
Source: Mol Ther Oncol. 2026 Apr 13;34(2):201205. doi: 10.1016/j.omton.2026.201205 (PMC13136644; doi:10.1016/j.omton.2026.201205)
Supplement: Document S1. Figures S1–S6 and Tables S1–S3 [file mmc1.pdf]

OMTON, Volume *34*

Supplemental information

NeuroD1 gene therapy inhibits glioma growth and extends life span through *in vivo* reprogramming approach

Yuchen Chen, Zuoyu Jiang, Sen Jin, Meng Liu, Ming Chen, Tsang-Chih Kuo, Kai Zhou, Liting Pu, Ming Chen, Shiyuan Chen, Xuetao Li, Adalia S. Chen, Jingmu Xie, Huitao Zhang, Qingsong Wang, Jie Xu, Jian Sheng, Yulun Huang, and Gong Chen

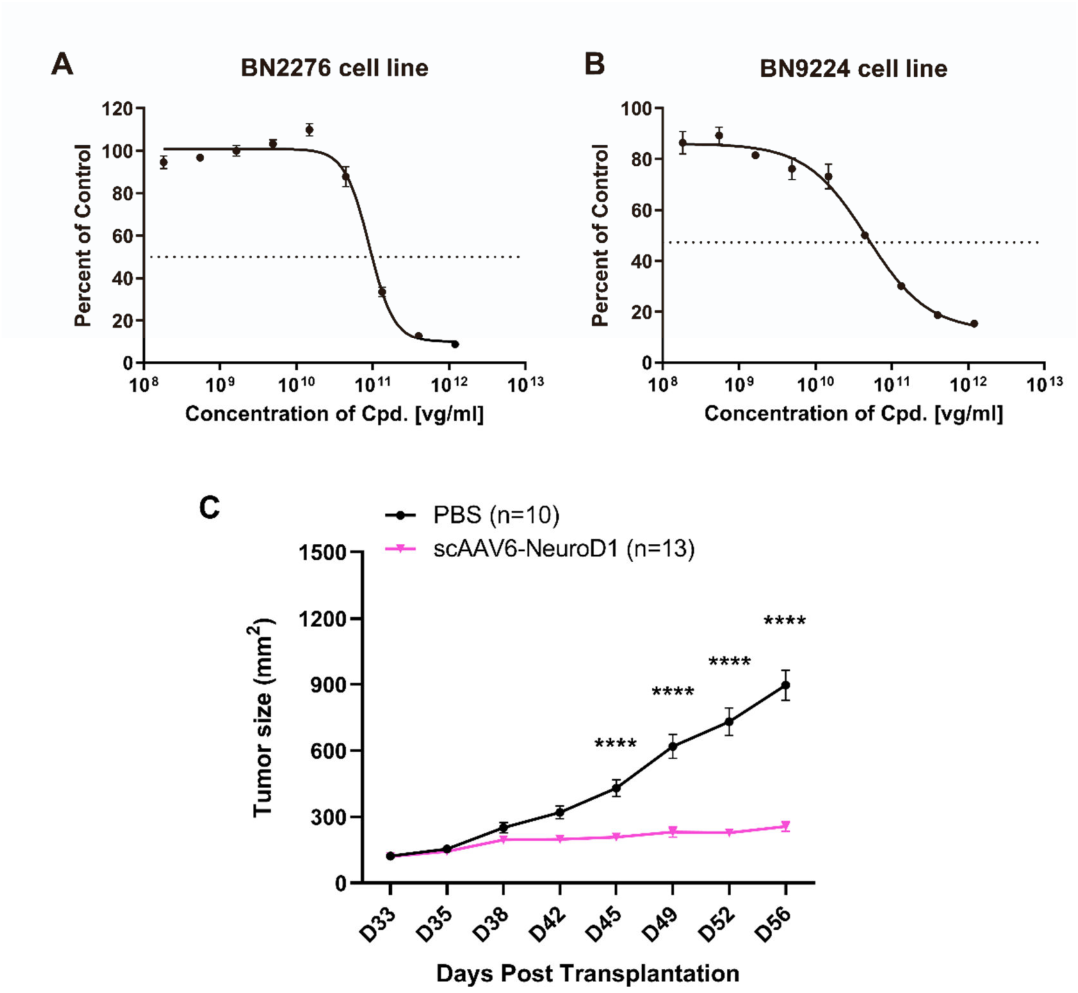

Figure S1. In Vitro and In Vivo Efficacy of scAAV6-NeuroD1 in PDX Models of Glioblastoma

(A-B) Dose-response curves illustrating the effect of scAAV6-NeuroD1 on cell viability in patient-derived glioblastoma cell lines BN2276 (A) and BN9224 (B). Cells were treated with increasing concentrations of scAAV6-NeuroD1, and viability was assessed using a CCK-8 assay. The IC50 values (dash line) were approximately 9.8×10^{11} vg/mL for BN2276 and 4.6×10^{10} vg/mL for BN9224. Data are presented as mean ± SEM (n=3 per group).

(C) Tumor growth curves in subcutaneous BN2276 glioblastoma PDX model bearing BN2276 treated with scAAV6-NeuroD1 or PBS (control). Tumor size was measured

at regular intervals post-transplantation. scAAV6-NeuroD1 treatment significantly inhibited tumor growth compared to the control group, with a marked reduction in tumor size observed by Day 56 post-transplantation. Data are presented as mean ± SEM (n=8-10 per group). ****$p<0.0001$ by two-way ANOVA followed by Tukey's post hoc test.

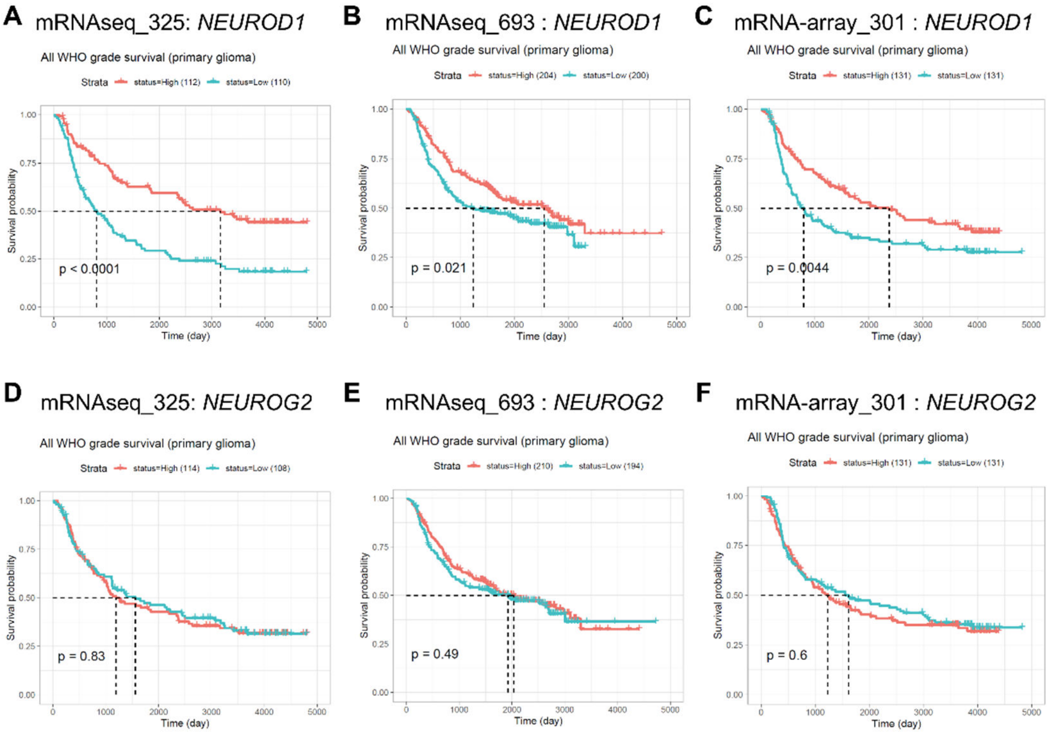

Figure S2. NeuroD1 but not Neurog2 expression positively correlated with survival in primary glioma from three CGGA cohorts.

Overall survival data of *NEUROD1* and *NEUROG2* in glioma patients from three CGGA cohorts (mRNAseq_325, mRNAseq_693 and mRNA-array_301) (**A-F**). The *NEUROD1* and *NEUROG2* expression levels were classified into low and high groups by CGGA website. Cohort: mRNAseq_325, n=222; cohort: mRNAseq_693, n=404; cohort: mRNA-array_301, n=262.

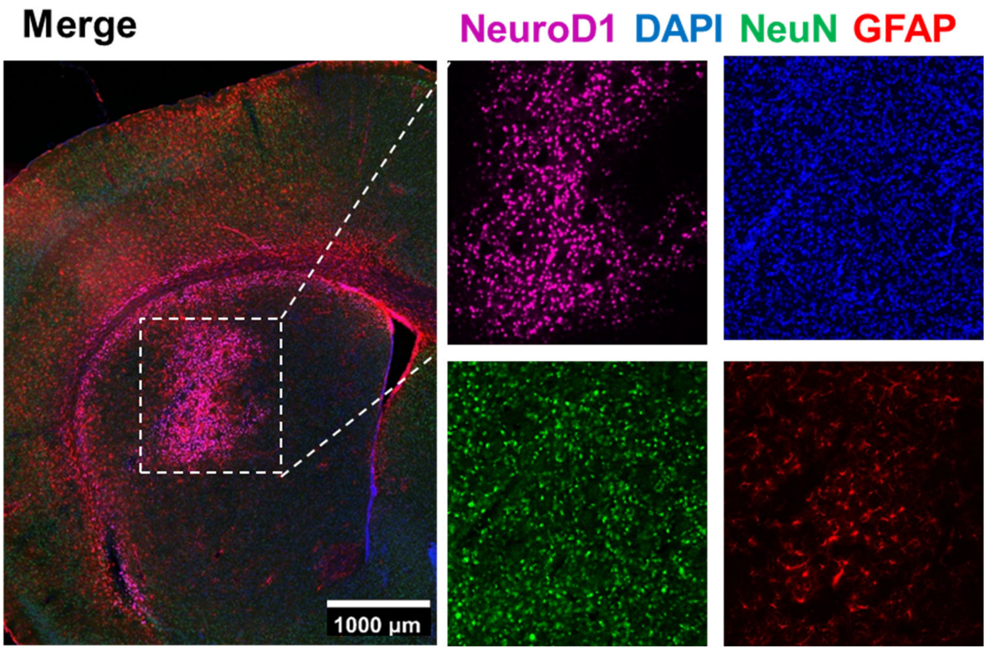

Figure S3. Transduction of scAAV6-NeuroD1 in healthy mouse brain.

Distribution of scAAV6-NeuroD1 and high magnification view of transduction of scAAV6-NeuroD1 in healthy mouse brain 3 days post-intracranial injection, showing NeuroD1 expression (magenta) in striatal areas without evident tissue disruption. Neuronal marker NeuN and DAPI marker display normal morphology and organization, with GFAP expression indicating no significant reactivity of astrocytes, indicating no significant pathological changes resulting from off-target AAV expression. Scale bar = 1000 µm.

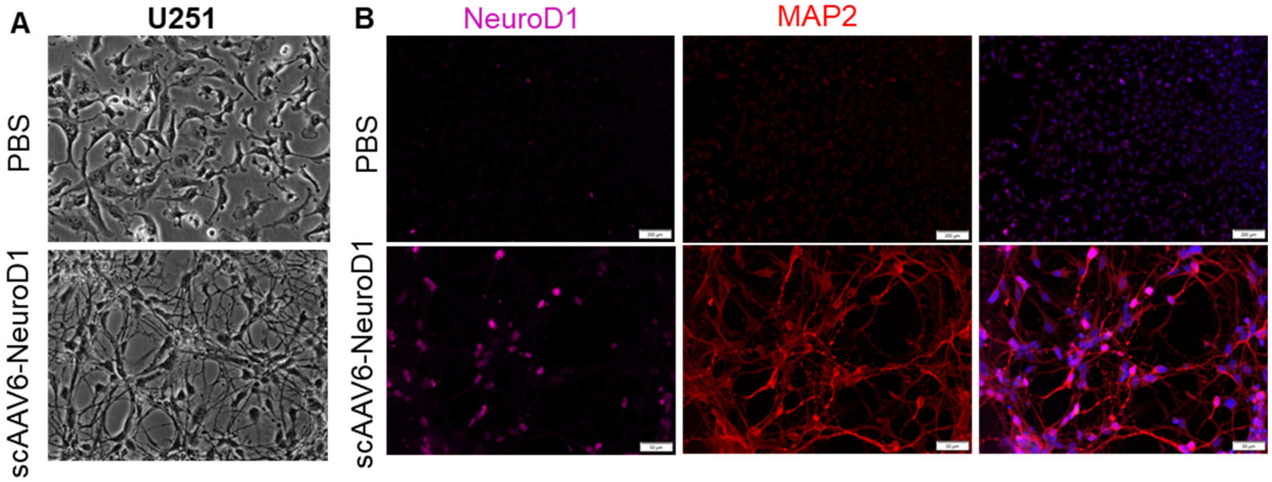

Figure S4. scAAV6-NeuroD1 induces neuronal conversion in U251 glioma cells in vitro.

(A) Phase-contrast images showing morphological changes in U251 cells following scAAV6-NeuroD1 transduction. scAAV6-NeuroD1-treated cells (lower panel) display neurite outgrowth and adopt a neuronal-like morphology at 14 days post infection.

(B) Immunofluorescence analysis of neuronal marker expression. scAAV6-NeuroD1-treated cells (bottom row) exhibit strong NeuroD1 (purple) and MAP2 (red) expression, confirming neuronal reprogramming. Nuclei are counterstained with DAPI (blue). Scale bar = 50 μm.

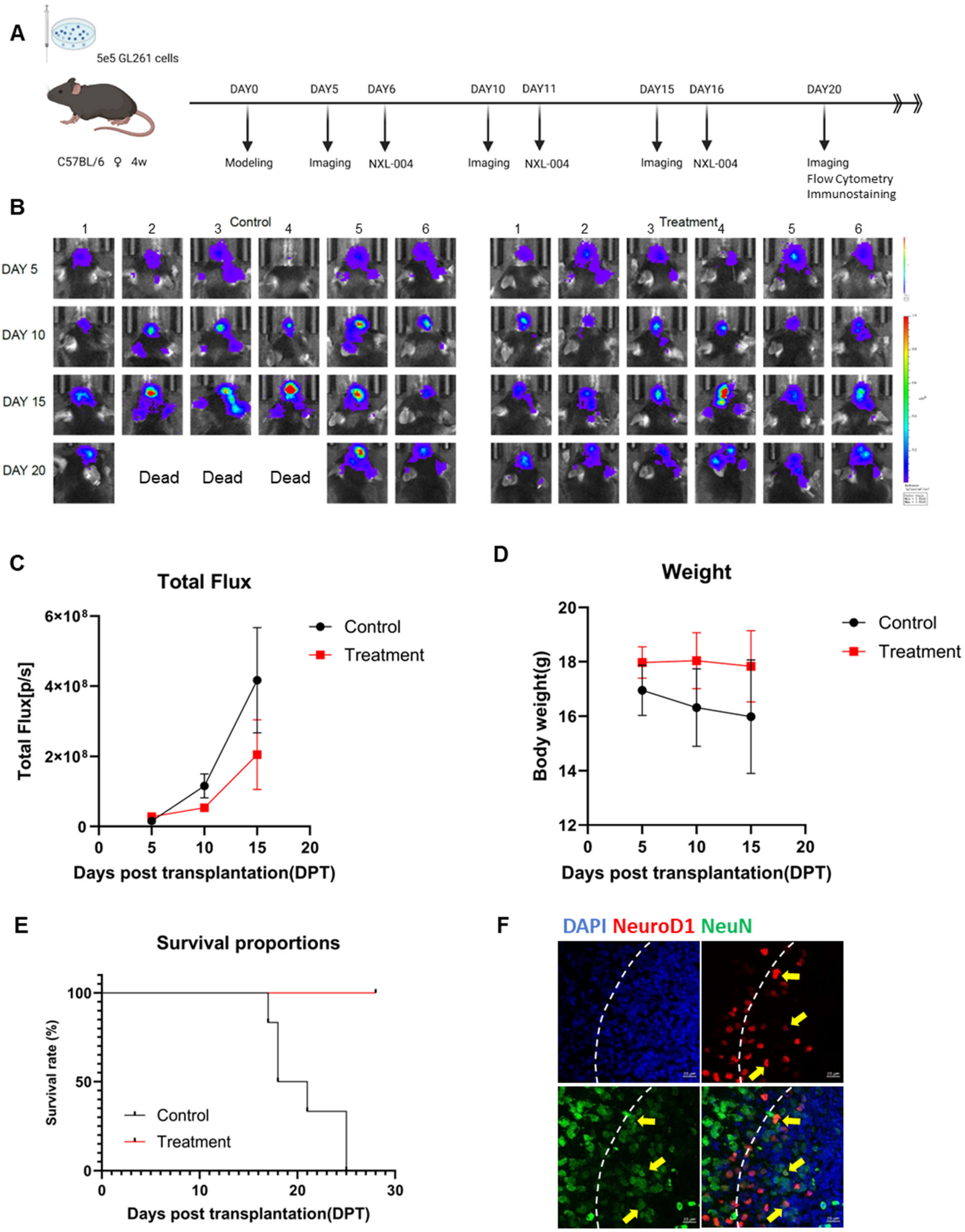
A
5e5 GL261 cells
C57BL/6 ♀ 4w
DAY0
DAY5
DAY6
DAY10
DAY11
DAY15
DAY16
DAY20
Modeling
Imaging
NXL-004
Imaging
NXL-004
Imaging
NXL-004
Imaging
Flow Cytometry
Immunostaining
B
Control
Treatment
DAY 5
DAY 10
DAY 15
DAY 20
Dead
Dead
Dead
C
Total Flux
Total Flux[p/s]
Days post transplantation(DPT)
Control
Treatment
D
Weight
Body weight(g)
Days post transplantation(DPT)
Control
Treatment
E
Survival proportions
Survival rate (%)
Days post transplantation(DPT)
Control
Treatment
F
DAPI NeuroD1 NeuN

Figure S5. scAAV6-NeuroD1 treatment prolongs survival and enhances antitumor immunity in an orthotopic GL261 GBM mouse model.

(A) Schematic of the experimental timeline. C57BL/6 mice were intracranially implanted with 5×10^5 GL261-luc cells on day 0, followed by intratumoral injections of scAAV6-NeuroD1 or PBS (control) on 5-, 10-, and 15-days post transplantation (DPT). Tumor progression was monitored periodically via IVIS (in vivo imaging).

(B) Representative bioluminescence images of mice from control and treatment groups at indicated days post-transplantation (DPT). Control mice showed progressive tumor growth and mortality by day 20, while the treatment group exhibited reduced tumor signals.

(C) Quantitative analysis of total flux (photons/sec) derived from bioluminescence imaging. The treatment group demonstrated a decrease in total flux compared to controls, indicating effective tumor suppression. Data are presented as mean ± SEM. $p = 0.1545$ at 15 DPT by Sidák's multiple comparisons test.

(D) Body weight changes of mice during the study period. The treatment group exhibited a less pronounced decline compared to controls as tumor progressed.

(E) Kaplan–Meier survival curves of control and treated groups (n=6 per group). The treatment group demonstrated a significantly higher survival rate, highlighting the therapeutic benefit of scAAV6-NeuroD1. $p<0.001$ by Log-rank (Mantel-Cox) test.

(F) Representative immunofluorescence image of NeuroD1 (red) and NeuN (green) at 28 days post-tumor transplantation (DPT). The image shows the border region between tumor tissue (right) and normal brain tissue (left), indicated by dense DAPI staining (blue) on the right. NeuroD1 expression (red) is observed along the tumor boundary, with partial co-localization with the neuronal marker NeuN (green), as indicated by yellow arrows. These findings suggest neuronal reprogramming at the tumor–normal interface. Scale bar = 20 µm.

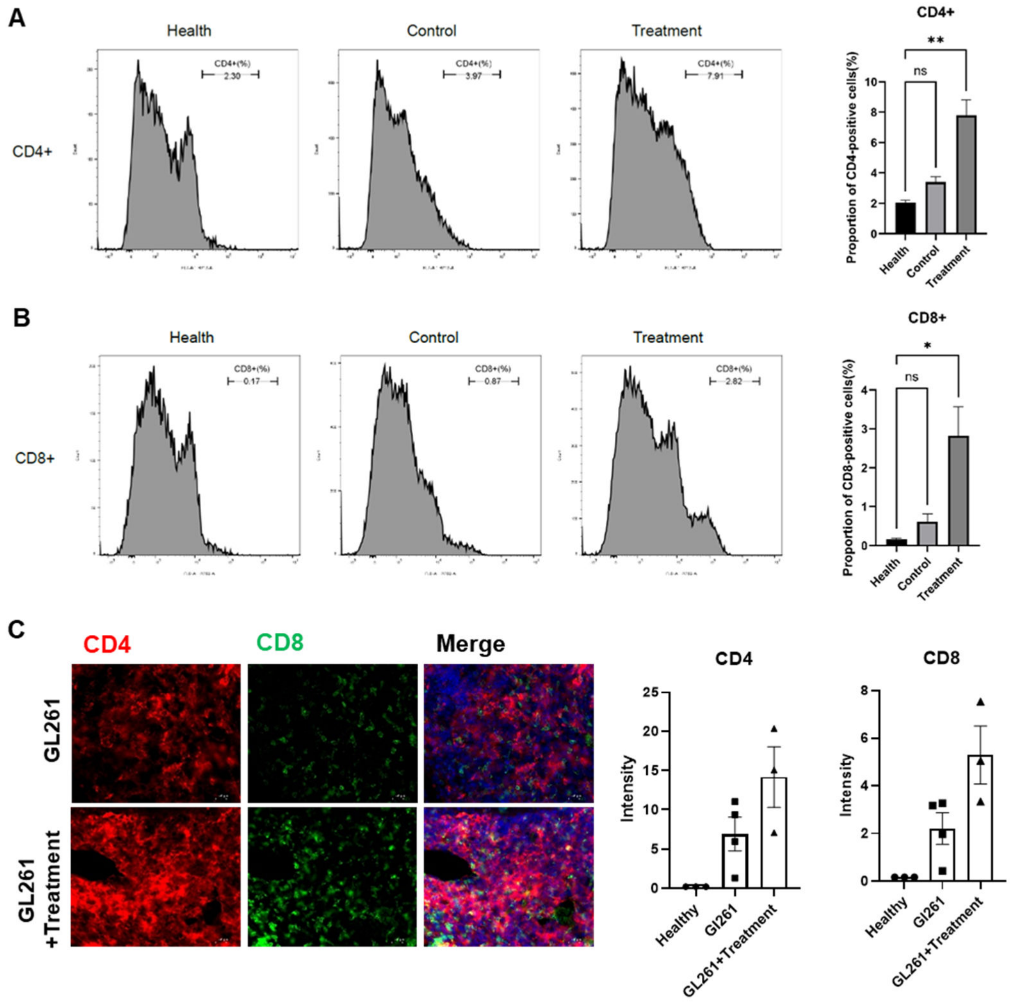

Figure S6. Analysis of T cell infiltration in brain tissues upon scAAV6-NeuroD1 treatment in GL261 GBM mouse model.

(A) Flow cytometry analysis of $CD4^+$ T cell infiltration in brain tissues from healthy, tumor-bearing control, and treated mice. Representative flow plots (left) and quantitative summary (right) show the percentage of $CD4^+$ T cells among $CD45^+$ immune cells.

(B) Flow cytometry analysis of $CD8^+$ T cell infiltration under the same conditions. Representative flow plots (left) and quantitative data (right) display the proportion of $CD8^+$ T cells.

(C) Immunofluorescence staining of $CD4^+$ (red) and $CD8^+$ (green) T cells in brain sections, with DAPI (blue) indicating nuclei. Merged images and quantitative fluorescence intensity analysis further confirm T cell recruitment into the tumor region.

Data are presented as mean ± SEM; $*p < 0.05$, $**p < 0.01$ by ordinary one-way ANOVA with Tukey's post hoc test.

Table S1. Clinicopathologic characteristics of patients with high and low expression of NEUROD1 in primary glioma (CGGA cohort: mRNAseq_325)

Table S1			
**Characteristic**	**Low *NEUROD1* expression**	**High *NEUROD1* expression**	**P value**
No. of patients	110[@]	112[@]	
Gender			
Female	39	45	0.4681[a]
Male	71	67	
WHO grade			
II+III	52	85	<0.0001[a]
IV	58	27	
IDH status			
Wild type	74	35[#]	<0.0001[a]
Mutation	36	76[#]	

[@]Grouping followed CGGA survival data.
[#]Patient had no *IDH* status record.
[a]P values were determined by chi-square test.

Table S2. Clinicopathologic characteristics of patients with high and low expression of *NEUROD1* in primary glioma (CGGA cohort: mRNAseq_693)

Table S2			
**Characteristic**	**Low *NEUROD1* expression**	**High *NEUROD1* expression**	**P value**
No. of patients	200[@]	204[@]	
Gender			
Female	81	92	0.3504[a]
Male	119	112	
WHO grade			
II+III	120	151	0.0027[a]
IV	80	53	
IDH status			
Wild type	102[#]	68[#]	<0.0001[a]
Mutation	72[#]	125[#]	

[@]Grouping followed CGGA survival data.
[#]Patients had no *IDH* status records.
[a]P values were determined by chi-square test.

Table S3. Clinicopathologic characteristics of patients with high and low expression of *NEURODI* in primary glioma (CGGA cohort: mRNA-array_301)

Table S3.			
**Characteristic**	**Low *NEUROD1* expression**	**High *NEUROD1* expression**	**P value**
No. of patients	131[@]	131[@]	
Gender			
Female	48	58	0.2081[a]
Male	83	73	
WHO grade			
II+III	66	89	0.0038[a]
IV	65	42	
IDH status			
Wild type	86[#]	60[#]	0.0012[a]
Mutation	44[#]	70[#]	

[@]Grouping followed CGGA survival data.
[#]Patients had no *IDH* status records.
[a]P values were determined by chi-square test.
