# Supplementary material for: NeuroD1 gene therapy inhibits glioma growth and extends life span through in vivo reprogramming approach
Source: Mol Ther Oncol. 2026 Apr 13;34(2):201205. doi: 10.1016/j.omton.2026.201205 (PMC13136644; doi:10.1016/j.omton.2026.201205)
Supplement: Document S2. Article plus supplemental information [file mmc2.pdf]

Molecular Therapy
Oncology
Original Article

NeuroD1 gene therapy inhibits glioma growth and extends life span through *in vivo* reprogramming approach

Yuchen Chen,[1,5,6] Zuoyu Jiang,[2,5] Sen Jin,[1,5] Meng Liu,[1,5] Ming Chen,[1,5] Tsang-Chih Kuo,[1,5] Kai Zhou,[1,5] Liting Pu,[1] Ming Chen,[1] Shiyuan Chen,[1] Xuetao Li,[2] Adalia S. Chen,[1] Jingmu Xie,[3] Huitao Zhang,[3] Qingsong Wang,[3,4] Jie Xu,[1] Jian Sheng,[1,6] Yulun Huang,[2,6] and Gong Chen[1,3,6]

[1]NeuExcell Therapeutics, 218 Xinghu Street, Building B1, Floor 9, Suzhou, Jiangsu 215000, China; [2]Department of Neurosurgery, The Fourth Affiliated Hospital of Soochow University, Suzhou 215000, China; [3]Guangdong-Hong Kong-Macau Institute of CNS Regeneration (GHMICR), Jinan University, Guangzhou 510632, China; [4]Chinese Medicine Guangdong Laboratory, Hengqin, Guangdong 519000, China

Glioblastoma(GBM), a highly aggressive primary brain tumor characterized by rapid progression, frequent recurrence, and limited clinical options, remains one of the most lethal central nervous system malignancies. Here, we report a gene therapy strategy to treat glioma utilizing NeuroD1, a neurogenic transcription factor with demonstrated capacity to reprogram both glial cells and GBM cells into neuronal lineages. We developed a self-complementary adeno-associated virus (scAAV) vector, scAAV6-NeuroD1, and evaluated its therapeutic potential across *in vitro* and *in vivo* GBM models, including multiple GBM cell lines, patient-derived organoids, and orthotopic models in immunodeficient mice. Our findings reveal that scAAV6-NeuroD1 preferentially infects glioma cells and induces dual therapeutic effects by simultaneously inhibiting glioma cell proliferation and inducing neuronal reprogramming. Importantly, scAAV6-NeuroD1-treated mice with orthotopic GBM transplants exhibited reduced tumor burden, infiltration of T cells into the glioma, attenuated tumor-induced body weight loss, and dose-dependent survival extension. Analysis of published patient datasets further revealed that high NeuroD1 expression level correlates with improved overall survival and lower tumor malignancy grade. Together, these findings position scAAV6-NeuroD1 as a promising therapeutic candidate, potentially redefining the therapeutic landscape for GBM.

INTRODUCTION

Glioblastoma (GBM), classified as a World Health Organization (WHO) grade 4 primary brain tumor, remains one of the most lethal malignancies in adults,[1,2] characterized by diffuse infiltration, therapeutic resistance, and dismal prognosis.[3] Despite multimodal therapies—including maximal surgical resection, radiotherapy, and temozolomide (TMZ)-based chemotherapy—the median overall survival rarely exceeds 15–18 months,[4–6] with nearly universal recurrence driven by residual-therapy-resistant cells.[1] This inevitable recurrence and lack of durable treatment underscore the urgent need for mechanistically innovative strategies to combat GBM's aggressiveness.

Recent advancements in cellular reprogramming have opened new avenues for treating CNS (central nervous system) disorders, particularly leveraging the plasticity of glial cells. One promising approach involves NeuroD1-mediated direct reprogramming of astrocytes to functional neurons (AtN),[7–10] which has shown to restore neural circuits and functions in preclinical models of ischemic stroke,[11] Huntington disease,[12] epilepsy,[13] spinal cord injury,[14] and Alzheimer disease.[10] Building on this concept, researchers including our team have demonstrated that neurogenic transcriptional factors (e.g., NeuroD1 and Neurogenin2) and small molecule cocktails not only reprogram glioma cells into neuron-like cells but also suppress their proliferative and invasive phenotypes.[15–23] This dual functionality—exploiting GBM's glial origin while subverting its malignancy—offers a groundbreaking therapeutic avenue of this challenging malignancy. However, translating these findings into clinical applications has been hindered by the absence of safe, efficient delivery systems capable of targeting invasive tumor niches.

Adeno-associated virus (AAV) vectors, renowned for their low immunogenicity and high transduction efficiency, have emerged as a promising delivery system for CNS gene therapy.[24–26] In oncology,

Received 14 May 2025; accepted 9 April 2026;
https://doi.org/10.1016/j.omton.2026.201205.

[5]These authors contributed equally

[6]These authors contributed equally

Correspondence: Yuchen Chen, NeuExcell Therapeutics, 218 Xinghu Street, Building B1, Floor 9, Suzhou, Jiangsu 215000, China.
E-mail: ycchen@neuexcell.com
Correspondence: Yulun Huang, The fourth Affiliated Hospital of Soochow University, No.9 Chongwen Road, Suzhou, Jiangsu 21500, China.
E-mail: huangyulun@suda.edu.cn
Correspondence: Gong Chen, NeuExcell Therapeutics, 218 Xinghu Street, Building B1, Floor 9, Suzhou, Jiangsu 215000, China.
E-mail: gongchen@neuexcell.com

Published by Elsevier Inc. on behalf of The American Society of Gene and Cell Therapy.
This is an open access article under the CC BY license (http://creativecommons.org/licenses/by/4.0/).

AAV-based strategies, such as delivery of tumor-suppressive genes or immunomodulators, have shown promise in GBM models.[27–31] Here, we engineered a self-complementary AAV6 vector (scAAV6) encoding NeuroD1 to evaluate its therapeutic potential against GBM. Our data revealed that scAAV6-NeuroD1 effectively inhibited GBM growth in both *in vitro* and *in vivo* models, including GBM cell lines, patient-derived organoids, and orthotopic CDX (cell-line-derived xenograft) mouse models. Additionally, scAAV6-NeuroD1 significantly prolonged survival in the CDX model in a dose-dependent manner while exhibiting synergistic effect with TMZ. These results establish scAAV6-NeuroD1 as a translatable candidate for clinical trials, supporting further clinical development for GBM treatment.

RESULTS

Screening of AAV serotypes for high-efficiency transduction and gene delivery to glioma cells

Our previous studies, along with others, have demonstrated that single neural transcription factors, including NeuroD1, Ascl1, and Neurog2, can convert GBM cells into post-mitotic neuron-like cells, significantly suppressing tumor proliferation.[15–17] While retroviral/lentiviral systems have been commonly used in proof-of-concept studies, their clinical translation faces challenges due to limited transduction efficiency and low expression level. To address this, we developed an AAV-based NeuroD1 delivery platform, leveraging the clinical success of AAV vectors in neurological disorders.[27] To optimize NeuroD1 delivery in GBM, we compared different AAV serotypes (6 vs. 9) and promoters (GFAP vs CMV) using green fluorescent protein (GFP) as a reporter. Four vectors were constructed: ssAAV9-GFAP::GFP (single-strand), ssAAV6-GFAP::GFP, scAAV9-CMV::GFP (self-complementary), and scAAV6-CMV::GFP. The rationale for vector design integrates two key considerations: (1) serotype: while AAV9 has achieved broad preclinical-to-clinical adoption for nervous tissue targeting,[25] AAV6 demonstrates efficient infection toward glioma[32] and was successfully deployed to deliver CXCL9 for GBM immunomodulation[33]; (2) promoter selection: glial-specific GFAP promoter and constitutively active CMV promoter are compared to balance cellular specificity with robust transgene expression.

U87-MG cell line was utilized to test transduction efficiency and GFP intensity at 24, 48, and 72 h after infection (Figure 1A). Quantitative analysis showed that scAAV6-CMV::GFP achieved significantly faster and higher expression of GFP (Figure 1B). Based on this, we engineered scAAV6-NeuroD1 with CMV promoter as our vector for subsequent studies in GBM models. The *in vitro* expression of NeuroD1 of scAAV6-NeuroD1 was verified with immunostaining in U87-MG cell lines (Figure 1C).

We then performed cell viability and proliferation assays in three different GBM cell lines (U87-Luc, U251-Luc, and GL261-Luc) to evaluate the effects of scAAV6-NeuroD1. Quantitative analysis in Figure 2A showed that scAAV6-NeuroD1 induced dose-dependent inhibition of cell viability across all cell lines. The inhibitory effects were most pronounced in U87-Luc and U251-Luc cells, showing 60%–75% viability decrease at 1×10^6 (1E6) MOI. Importantly, the AAV6 empty vector showed negligible impact on cell viability, confirming the NeuroD1-specific therapeutic effects. Furthermore, scAAV6-NeuroD1 significantly suppressed U87-Luc sphere formation by over 90% compared to both PBS control and scAAV9-CMV::NeuroD1 (Figures 2B and 2C), demonstrating its anti-proliferative and anti-tumorigenic effects. Interestingly, while inhibition of proliferation was observed in all three cell lines, only U251 demonstrated conversion toward neuronal morphology along with expression of neuronal marker Map2 (Figure S4), consistent with our previous study.[16] These results indicate that the capability of glioma cells to undergo *trans*-differentiation depends on cell intrinsic properties.

Collectively, we engineered an scAAV serotype 6 vector with the CMV promoter to deliver NeuroD1, which potently suppressed tumor cell proliferation and sphere formation *in vitro*, indicating its potential as a therapeutic candidate for GBM.

scAAV6-NeuroD1 promoted glioma-to-neuron conversion in GBM organoid and induced apoptosis

Patient-derived glioma organoids have emerged as a powerful preclinical model for evaluating treatment efficacy in patient-specific context.[34–36] To assess the translational potential of scAAV6-NeuroD1, we treated GBM organoid with scAAV6-NeuroD1 *in vitro* using MOI gradient (multiplicities of infection: 1E5, 1.5E6, and 5E6) at a virus titer of 1×10^{13} vg/mL. Following a 14-day incubation period, the organoid samples were processed for histopathological analysis, including hematoxylin and eosin (H&E) staining and immunofluorescent staining. As shown in Figures 3A and 3B, the untreated organoid in the control group maintained intact spherical morphology with dense DAPI+ nuclei (blue) and uniform expression of glioma marker GFAP (red) but lacked neuronal signal NeuN (green).

Low-dose treatment induced detectable peripheral NeuroD1 expression (magenta, Figure 3B), suggesting penetration of vector from the superficial cell population. Notably, early structural dissociation became evident, as shown in H&E staining and DAPI staining (Figures 3A and 3B). In the mid-dose treatment group, NeuroD1 showed robust pan-organoid expression, accompanied by more obvious disruption of structural integrity (Figures 3A and 3B). Notably, neuronal marker NeuN emerged, while GBM marker GFAP decreased to 28.9% of the control level (Figure 3B). Significantly, high-dose treatment induced near-complete tissue dissociation (Figure 3A, bottom row). Within the remaining tissues, strong NeuN signal was detected (86.6-fold increase compared to control), with about 70% reduction in GFAP signal (Figure 3B, quantified in Figures 3C and 3D). Paradoxically, NeuroD1 expression decreased in high-dose group, likely attributable to MOI-dependent cytotoxicity (Figure 3B, quantified in Figure 3E).

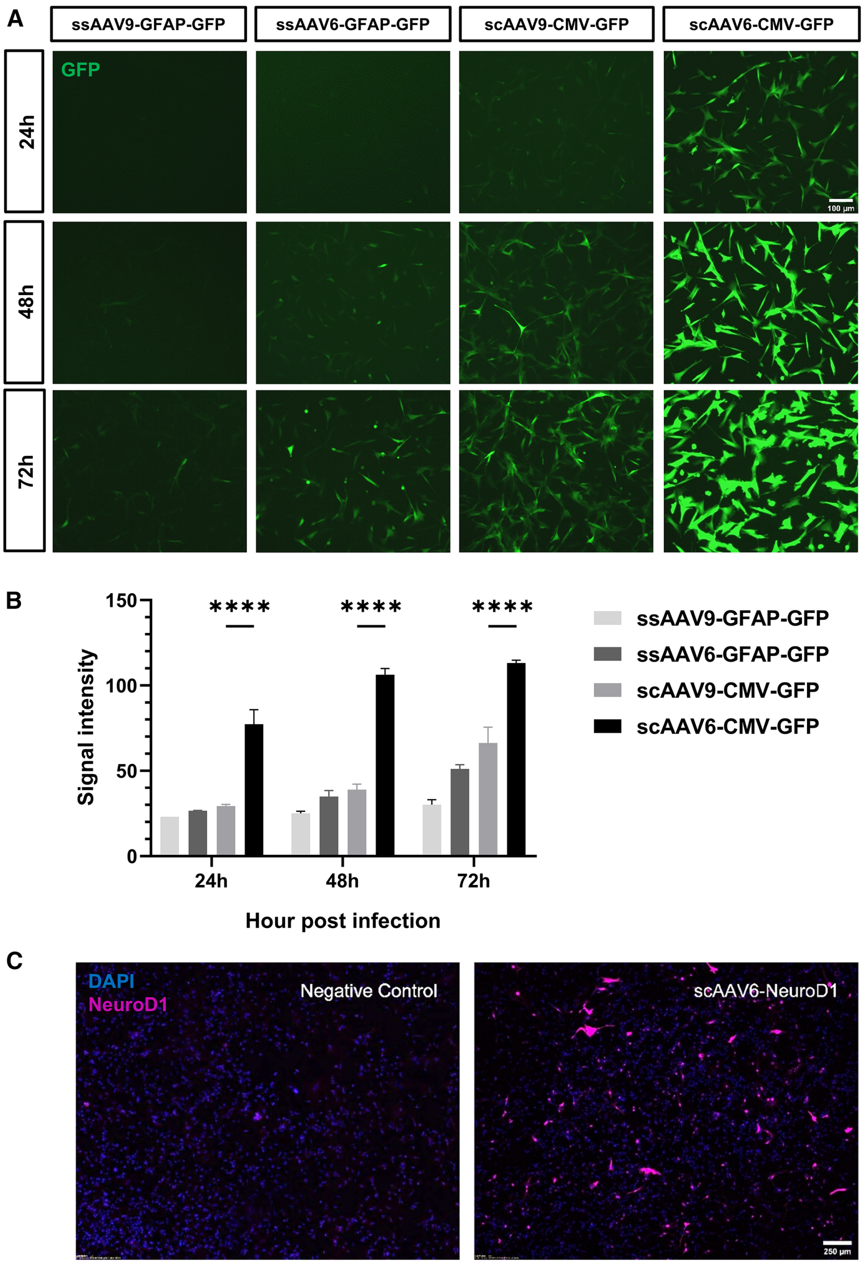

Figure 1. Optimization of AAV serotype and promoter for efficient gene delivery in U87-MG cells
(A) Representative fluorescence images showing the expression of GFP in U87-MG cells at 24, 48, and 72 h post-infection with various AAV vectors: ssAAV9-GFAP::GFP, ssAAV6-GFAP::GFP, scAAV9-CMV::GFP, and scAAV6-CMV::GFP. The scAAV6-CMV::GFP vector exhibited the strongest and most rapid GFP expression, indicating superior transduction efficiency. Scale bars, 100 μm. (B) Quantification of GFP fluorescence intensity at 24, 48, and 72 h post-infection. scAAV6-CMV::GFP vector showed significantly higher fluorescence intensity compared to the other vectors at all time points, as determined by one-way ANOVA followed by Tukey's post hoc test (****$p < 0.0001$). Data are presented as mean ± SEM from three independent experiments. (C) Immunofluorescence images of U87-MG cells transduced with scAAV6-CMV::NeuroD1 and stained for NeuroD1 (magenta) and DAPI (blue) at 72 h post-infection. scAAV6-CMV::NeuroD1 demonstrated robust NeuroD1 expression, confirming the vector's effectiveness in delivering the therapeutic gene.

RNA sequencing (RNA-seq) of high-dose group at 14 days post-infection revealed profound transcriptional reprograming: neuronal pathways including synaptogenesis (28 genes), glutamatergic signaling (27 genes), neurite outgrowth (22 genes), and synaptic transmission (18 genes) were significantly upregulated at least 2-fold (Figure 3F). Compared to the control, scAAV6-NeuroD1 induced 118.9-fold increase of *NEUROD1* expression, alongside marked upregulation of neurogenesis marker *DCX* (7.8-fold), synaptic regulator *SYN1* (2.5-fold), glutamatergic marker *CAMK2B* (1.8-fold), and neuronal maturation maker *NEUN* (1.9-fold). Concurrently, GBM-associated genes were downregulated, including *MKI67* (52% reduction), *GFAP* (85% reduction), *EGFR* (21% reduction), and *VIM* (22% reduction). These changes are indicative of a robust neurogenic response and alterations in cellular architecture induced by scAAV6-NeuroD1 treatment.

Given the extensive cellular dissociations observed, we further assessed apoptotic cell death through TUNEL (terminal deoxynucleotidyl transferase dUTP nick end labeling) assay. The results showed robust activation of apoptosis across all treatment groups (Figures 4A and 4B). Notably, organoids treated at high MOI doses (≥5E6) underwent significant tissue dissociation (Figures 4C and 4D).

Collectively, scAAV6-NeuroD1 demonstrates tripartite therapeutic effects on GBM patient-derived organoid via (1) activating neuronal pathways, (2) suppressing GBM genes, and (3) activating cytotoxic effects, positioning it as a multifaceted candidate for GBM treatment.

scAAV6-NeuroD1 significantly inhibits GBM growth and reduces tumor cell proliferation in the orthotopic CDX model

Following the discovery of scAAV6-NeuroD1's therapeutic effects *in vitro*, we next evaluated its efficacy *in vivo* in orthotopic GBM model. U87-MG cells were intracranially implanted into the striatum of immunodeficient nude mice, followed with intratumoral administration of scAAV6-NeuroD1 at 3, 8, and 13 days post-transplantation (DPT). The dosing schedule was designed based on our previous

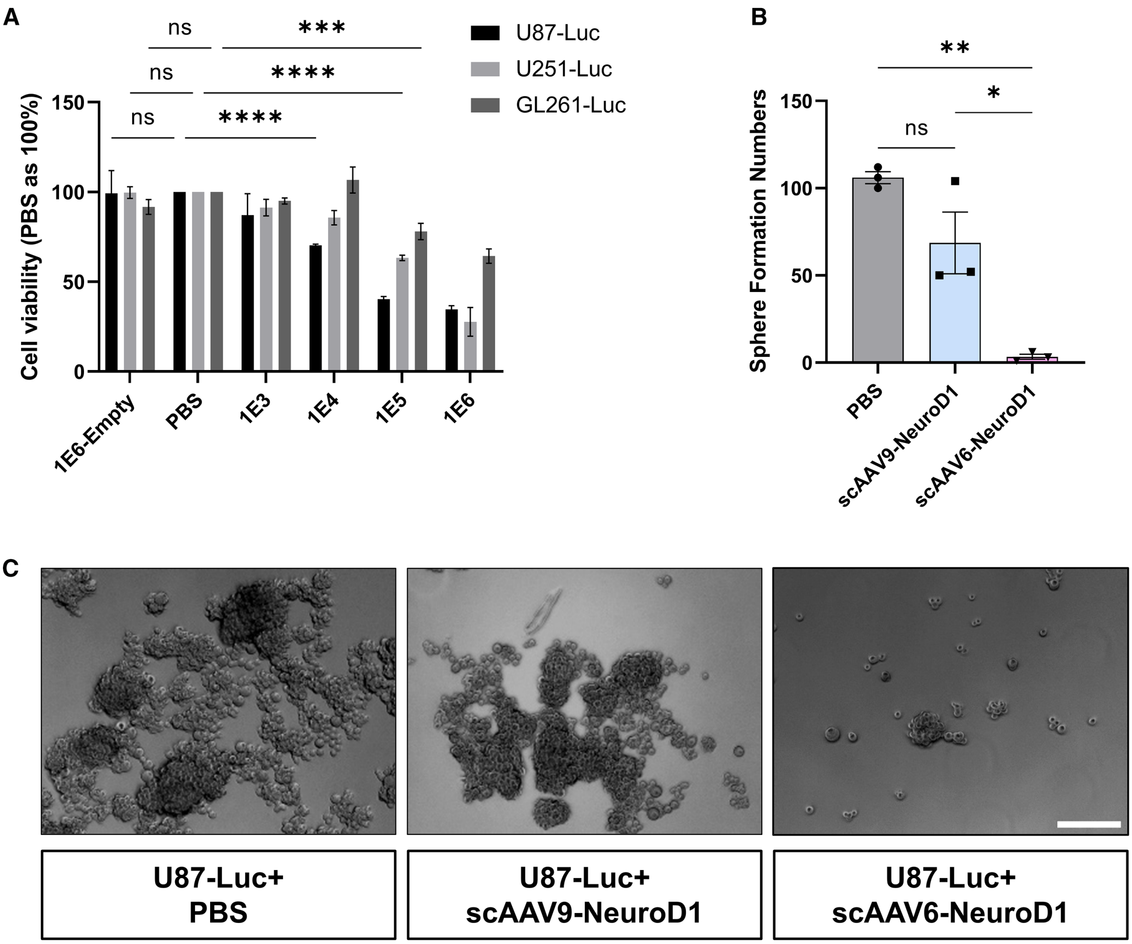

Figure 2. scAAV6-NeuroD1 inhibits glioblastoma cell proliferation and sphere formation *in vitro*

(A) Dose-dependent inhibition of cell viability in three glioblastoma (GBM) cell lines (U87-Luc, U251-Luc, and GL261-Luc) following treatment with scAAV6-NeuroD1. Cells were treated with increasing multiplicities of infection (MOIs: 1E3–1E6) of scAAV6-NeuroD1 or controls (PBS and AAV6 empty vector), and cell viability was assessed using the CCK-8 assay. Data are presented as mean ± SEM from three independent experiments. Statistical significance was determined using one-way ANOVA followed by Tukey's post hoc test. Significant reductions in cell viability were observed at higher MOIs of scAAV6-NeuroD1 in all three cell lines. $^{***}p < 0.001$, $^{****}p < 0.0001$. (B) Quantification of sphere formation in U87-Luc cells following treatment with scAAV6-NeuroD1 at an MOI of 1E6, compared to PBS control and scAAV9-NeuroD1. Data are shown as mean ± SEM. scAAV6-NeuroD1 treatment significantly reduced sphere formation, indicating impaired self-renewal capacity of GBM cells. (C) Representative brightfield images of U87-Luc spheres at 72 h post-treatment with PBS (control), scAAV9-NeuroD1, and scAAV6-NeuroD1 at MOI 1E6. scAAV6-NeuroD1 treatment led to a marked reduction in sphere size and number, supporting its inhibitory effects on GBM cell proliferation and tumorigenicity. Scale bars, 100 μm.

finding[37] that intracerebral redosing within 14 days minimizes neutralizing antibody induction, thereby maximizing the efficacy of repeated AAV delivery.

Bioluminescence imaging was performed using the *in vivo* imaging system (IVIS) at 2 DPT prior to therapeutic intervention to confirm successful establishment of the glioblastoma (GBM) transplant model in mouse brains. DAPI and H&E staining at 14 DPT revealed significantly smaller tumor size in treated mice compared to controls (Figures 5A and 5B). Untreated animals exhibited dense DAPI+ nuclei with defined brain-tumor edge, whereas scAAV6-NeuroD1-treated-tumor exhibited reduced nuclear density and disrupted tumor borders. Quantification confirmed an 81.5% tumor volume reduction in treated group compared to the controls (Figure 5C, $^{***}p < 0.001$).

To dynamically monitor tumor progression, bioluminescence was measured via IVIS at 2, 8, and 14 DPT with the same treatment protocol of scAAV6-NeuroD1 at 3, 8, and 13 DPT (Figure 5E). While both groups exhibited progressive signal increase, the treated group demonstrated significantly attenuated intensification compared to controls. Luminescent signals emerged as early as 2 DPT for all animals, but by 8DPT (right before the second treatment), treated mice showed 95.8% lower average radiance than the control group,

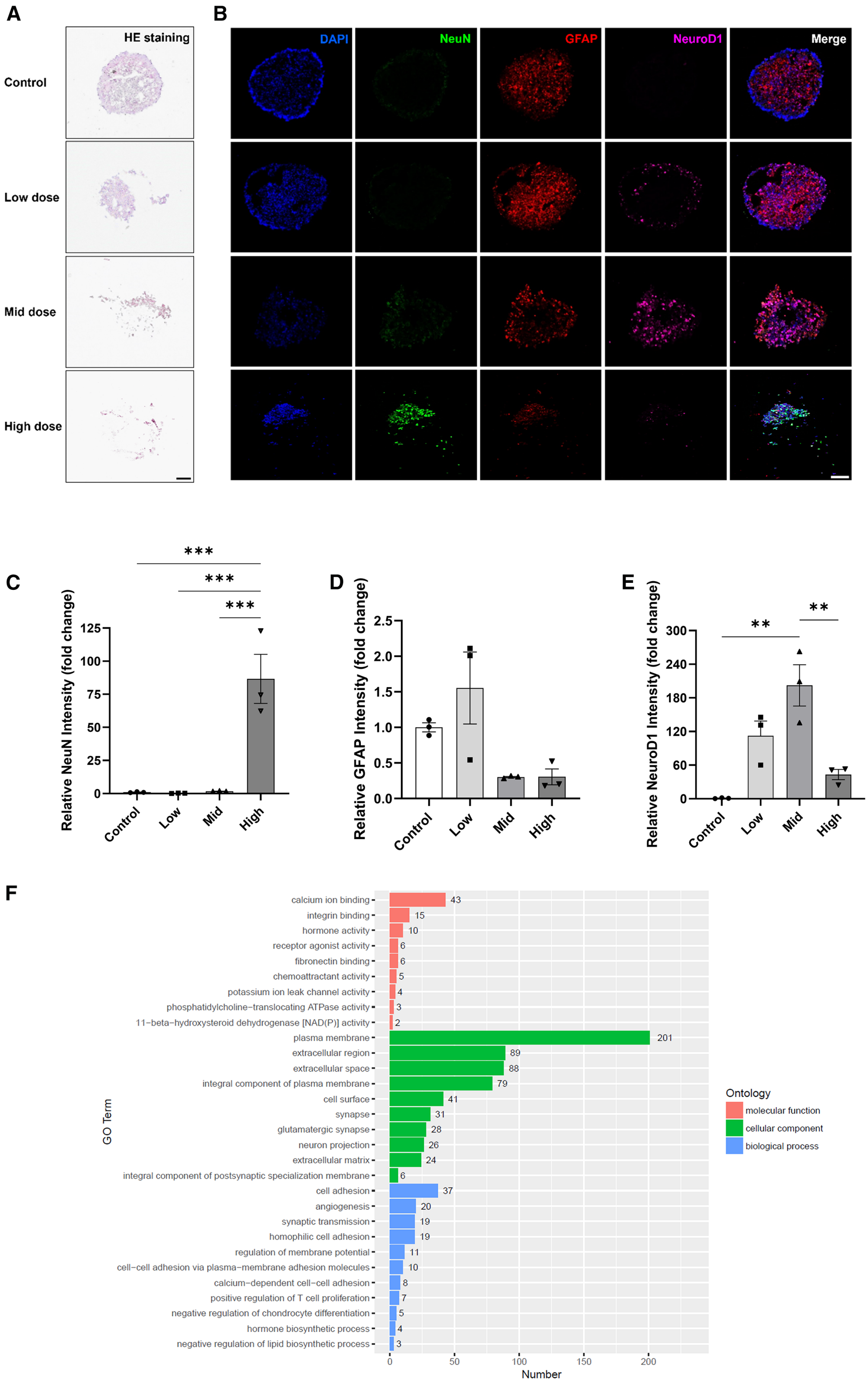

(legend on next page)

indicating early suppression of tumor growth. Additionally, scAAV6-NeuroD1 suppressed tumor proliferative activity, as evidenced by immunostaining and transcriptional downregulation of Ki67. Treated tumors showed significantly fewer Ki67+ cells in immunostaining and 85.6% reduced level of *MKI67* mRNA, compared to the controls (Figures 5D–5F). These data established that scAAV6-NeuroD1 reduces tumor size via effectively inhibiting tumor cell proliferation in orthotopic U87-MG mouse model.

scAAV6-NeuroD1 drives glioma cell neuronal lineage transformation and activates microglia/macrophage-mediated innate immunity *in vivo*

Following the observed tumor reduction with scAAV6-NeuroD1, we investigated the underlying mechanisms in U87-MG glioblastoma models. Specifically, we aimed to determine whether scAAV6-NeuroD1 promotes glioma-to-neuron conversion and activates tumor-related immune responses in the U87-MG CDX orthotopic model. Immunostaining revealed robust NeuroD1 expression selectively localized to the DAPI-dense tumor region as early as 24 h of the first treatment (Figure 6A), with negligible signal in adjacent brain tissue, confirming the tumor-selective tropism of scAAV6-NeuroD1 for GBM. By 14 DPT, DAPI-defined tumor region in control group maintained HuNu+ (human tumor cells) but lacked NeuroD1 or NeuN expression as expected (Figure 6B, top). In contrast, scAAV6-NeuroD1-treated group (Figure 6B, bottom two rows) exhibited sustained expression of NeuroD1 (confirmed by RT-qPCR; Figure 6C) and co-labeling of NeuN and NeuroD1 (Figure 6B, bottom). Consistent with prior observations, DAPI and HuNu signals were diffuse and lacked distinct tumor morphology in treated group. However, no clear co-localization of HuNu and NeuN signals was observed in these regions, potentially due to scAAV6-NeuroD1-induced glioma cell death combined with the inherent challenges of newborn neuron surviving in the glioma cell environment.

The activation of CNS-resident microglia and peripherally derived macrophage plays a crucial role in innate anti-tumor immunity. To evaluate microglia/macrophage activation, we performed immunostaining for the pan-macrophage lineage marker Iba1. Notably, at 14 DPT (one day after third treatment), scAAV6-NeuroD1-treated animals showed a marked increase in Iba1 signal intensity in the tumor area compared to controls, and these IBA1-positive microglia/macrophage are primarily located at the tumor margin and invasive fronts (Figures 6D and 6E). These results suggest that scAAV6-NeuroD1 inhibits tumor growth through multiple mechanisms, such as triggering neuronal lineage-associated changes in glioma cells and activating microglia/macrophage-mediated innate immune response, both of which likely contribute to its therapeutic efficacy in the U87 mouse model.

scAAV6-NeuroD1 delays body weight loss and extends lifespan in orthotopic GBM mouse model

To further evaluate the therapeutic impact of scAAV6-NeuroD1, we monitored body weight and survival in the U87MG-Luc orthotopic glioblastoma model with the same treatment protocol of scAAV6-NeuroD1 at 3, 8, and 13 DPT. Successful establishment of the orthotopic GBM model was confirmed through IVIS-based bioluminescence imaging performed at 2 DPT, prior to the administration of therapeutic interventions. Control animals exhibited over 10% body weight loss from 13 DPT onward (Figure 7A, black line), a hallmark of tumor progression. In contrast, scAAV6-NeuroD1-treated mice maintained stable body weight until 15 DPT (Figure 7A, purple line), indicating delayed disease progression.

Survival analysis demonstrated a dose-dependent increase in median survival across treatment groups (low-dose: 6×10^{11} vg/mL; mid-dose: 2×10^{12} vg/mL; high-dose 2×10^{13} vg/mL, 4 μL). Untreated controls ($n = 9$) and low-dose group ($n = 10$) showed equivalent median survival of 17 days post-tumor cell transplantation. Increased doses progressively extended survival: 24 days (Δ41%) for mid-dose group and 26 days (Δ53%) for high-dose group (Figure 7B). Strikingly, 20% of high-dose animals remained tumor-free at the 35-days endpoint, whereas all control mice succumbed by day 20. Dose stratification further confirmed efficacy gradient (low vs mid: $p = 0.0053$; mid vs high: $p = 0.008$; Figure 7B), confirming a progressive enhancement of therapeutic potency with escalating vector dosage.

We next tested scAAV6-NeuroD1 combined with temozolomide (TMZ), a frontline GBM chemotherapeutic agent. Monotherapy with scAAV6-NeuroD1 or TMZ achieved median survival of 27 days and 28 days, respectively, both surpassing controls (15.5 days). Notably, all TMZ-treated animals succumbed within 30 DPT, whereas 40% scAAV6-NeuroD1-treated animals survived tumor-free until 86 days study endpoint. Critically, the scAAV6-NeuroD1 plus TMZ combination completely prevented mortality throughout the 86-day observation period (Figures 7C and 7D), suggesting synergistic therapeutic effects.

Figure 3. scAAV6-NeuroD1 induced neuronal gene upregulation and GBM gene downregulation in organoid in a dose-dependent manner *in vitro*
(A) Hematoxylin and eosin (H&E) staining of glioblastoma (GBM) organoids following 14-day treatment with scAAV6-NeuroD1 at low, mid, and high multiplicities of infection (MOIs). The images demonstrate a dose-dependent increase in cellular dissociation, with high-dose treatment resulting in significant disruption of organoid structure. Scale bars, 200 μm (B) Immunofluorescence analysis of GBM organoids treated with scAAV6-NeuroD1 at varying doses. Organoids were stained with DAPI (blue) to mark nuclei, NeuN (green) as a neuronal marker, GFAP (red) as a glial marker, and NeuroD1 (magenta). The images reveal dose-dependent glia-to-neuron conversion, characterized by increased NeuN and NeuroD1 expression and decreased GFAP expression, particularly at higher doses. Scale bars, 200 μm. (C–E) Quantification of NeuN, GFAP, and NeuroD1 immunofluorescence intensity. Data are presented as mean ± SEM. $^{**}p < 0.001$, $^{***}p < 0.001$ by one-way ANOVA followed by Tukey's post hoc test. (F) Gene Ontology (GO) term enrichment analysis of differentially expressed genes in high-dose scAAV6-NeuroD1-treated GBM organoids at 14 days post-infection. The bar graph highlights significant upregulation of genes involved in synapse formation, synaptic transmission, neuron projection, and cell adhesion.

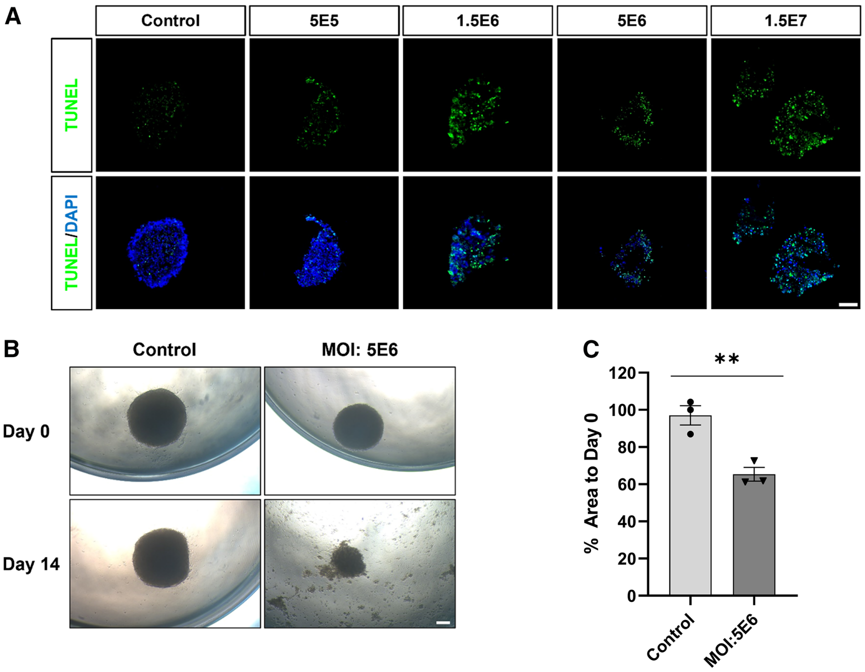

Figure 4. scAAV6-NeuroD1 promotes cell death in GBM organoids

(A) TUNEL assay of GBM organoids following 14-day treatment with scAAV6-NeuroD1 at increasing MOI gradient (5E5, 1.5E6, 5E6, and 1.5E7). Scale bars, 100 μm. (B) Representative bright-field images of GBM organoids treated with scAAV6-NeuroD1 (MOI: 5E6) versus untreated control. The experimental group showed obvious cell dissociation, loss of spherical structure, and cellular debris dispersion in extracellular matrix. Scale bars, 200 μm. (C) Quantitative analysis of the organoid size percentage at day 14 relative to that of day 0. Data are presented as mean ± SEM. ** $p < 0.01$ by unpaired two-tailed Student's *t* test.

In summary, scAAV6-NeuroD1 treatment delays tumor-associated body weight loss and dose-dependently extends survival in U87MG orthotopic GBM model, with combinatorial TMZ treatment achieving complete survival protection, highlighting its therapeutic potential in GBM treatment.

scAAV6-NeuroD1 exhibits robust anti-tumor efficacy and immune activation in immuno-competent orthotopic GBM models

Next, to better recapitulate the human GBM microenvironment, we used the GL261 immune-competent model to evaluate the therapeutic potential of scAAV6-NeuroD1. As outlined in Figure S5A, intracranial administration of scAAV6-NeuroD1 on days 6, 11, and 16 post-tumor implantation suppressed tumor progression, with bioluminescence imaging revealing reduced total flux in treated mice compared to controls (Figures S5B and S5C). Due to reduced tumor burden, scAAV6-NeuroD1 treatment attenuated tumor-associated weight loss (Figure S5D) and resulted in a marked survival extension (Figure S5E; $p < 0.001$), with the treatment group exhibiting a 100% survival at the experimental endpoint (28 DPT) while all control animals died within 25 days. Immunofluorescence analysis on day 28 revealed NeuroD1 expression along the tumor border and partial co-localization with the neuronal marker NeuN (Figure S5F). Furthermore, flow cytometry and immunofluorescence assay showed significantly enhanced recruitment of $CD4^+$ (7.91% vs. 3.97% in controls) and $CD8^+$ T cells (2.62% vs. 0.87% in controls) into the tumor microenvironment (Figure S6), highlighting the immune activation alongside the anti-tumor effects. Collectively, these results demonstrate robust anti-glioma efficacy of scAAV6-NeuroD1 within an immune-competent environment, supporting its clinical relevance.

scAAV6-NeuroD1 demonstrates robust anti-tumor activity in patient-derived xenograft models

Beyond CDX model, we evaluated the efficacy of scAAV6-NeuroD1 in two glioma-patient-derived xenograft (PDX) models (BN2276 and BN9224). *In vitro*, scAAV6-NeuroD1 induced a dose-dependent reduction of cell viability, with IC50 values of $\sim 9.8 \times 10^{11}$ vg/mL for BN2276 and $\sim 4.6 \times 10^{10}$ vg/mL for BN9224 (Figures S1A and S1B). *In vivo*, scAAV6-NeuroD1 was tested in NOD/SCID mice model bearing BN2276 subcutaneously, with anti-tumor efficacy quantified by longitudinal tumor volume measurement. Treated animals exhibited significant tumor growth suppression compared to the PBS controls, culminating in significantly smaller tumors by day 56 post-engraftment (Figure S1C). These data further validate the translational relevance of scAAV6-NeuroD1 across preclinical glioblastoma models, supporting its clinical translation as a promising GBM therapy.

High-level NeuroD1 expression correlated with improved survival of glioma patients

To explore the clinical implications of transcription factors in glioma, we further analyzed data from the TCGA-Lower Grade Glioma and Glioblastoma (GBMLGG) cohort (UCSC Xena: https://xena.ucsc.edu/) and three primary glioma cohorts from Chinese Glioma Genome Atlas (CGGA: http://www.cgga.org.cn/analyse/RNA-data.jsp).

The analysis showed that high level of *NEUROD1* expression was significantly associated with improved clinical outcomes, including prolonged overall survival (OS) and progression-free interval (PFI) (Figures 8A and 8B). In contrast, *NEUROG2*—another bHLH family of neural transcription factor paralog of *NEUROD1*—showed a nonsignificant trend toward improved survival (Figures 8C and 8D). These patterns were validated across multiple CGGA datasets (Figures S2A–S2F), confirming high *NEUROD1* expression linked to a better prognosis in glioma patients.

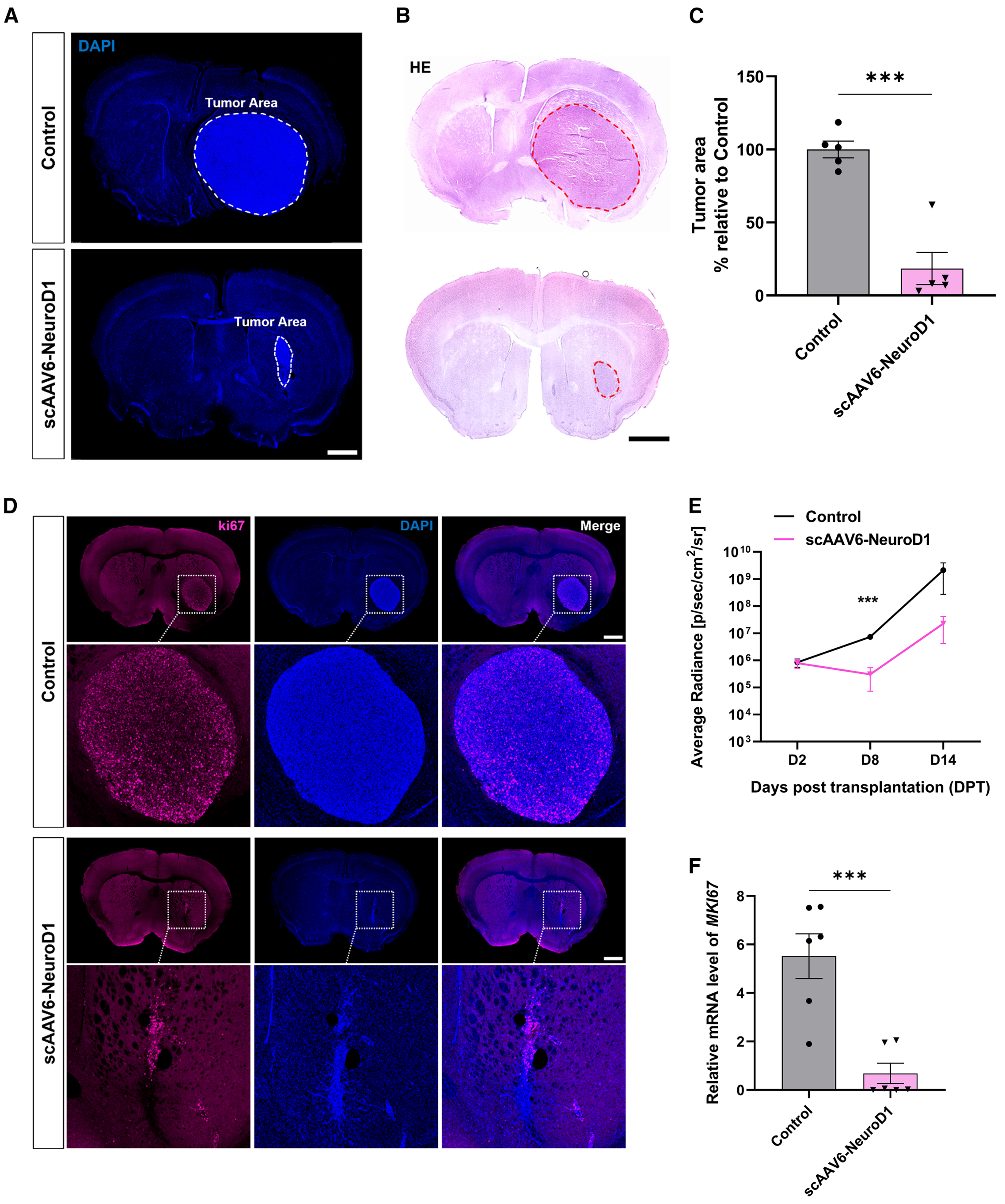

Figure 5. scAAV6-NeuroD1 reduces tumor size and inhibits cell proliferation in a U87 GBM orthotopic mouse model

Representative images of DAPI immunostaining (A) and hematoxylin and eosin (H&E) staining (B) comparing tumor area in control and scAAV6-NeuroD1-treated groups at 14 days post-tumor transplantation (DPT). Scale bars, 1,000 μm. (C) Quantification of tumor area using DAPI immunostaining images, showing a significant reduction in the scAAV6-NeuroD1-treated group relative to the control. Data are presented as mean ± SEM. ***$p < 0.001$ by unpaired two-tailed Student's *t* test. (D) Representative images of Ki67 immunostaining demonstrate reduced cell proliferation in the scAAV6-NeuroD1-treated group. Scale bars, 1,000 μm (E) Time course measurement of IVIS signals showing comparison of bioluminescent flux between control and scAAV6-NeuroD1-treated group. Data are presented as mean ± SEM (n = 5–6 per group). ***$p < 0.001$ by two-way ANOVA followed by Tukey's post hoc test. (F) RT-qPCR analysis of Ki67 mRNA expression confirms a significant decrease in proliferation markers in the scAAV6-NeuroD1-treated group. ***$p < 0.001$ by unpaired two-tailed Student's *t* test.

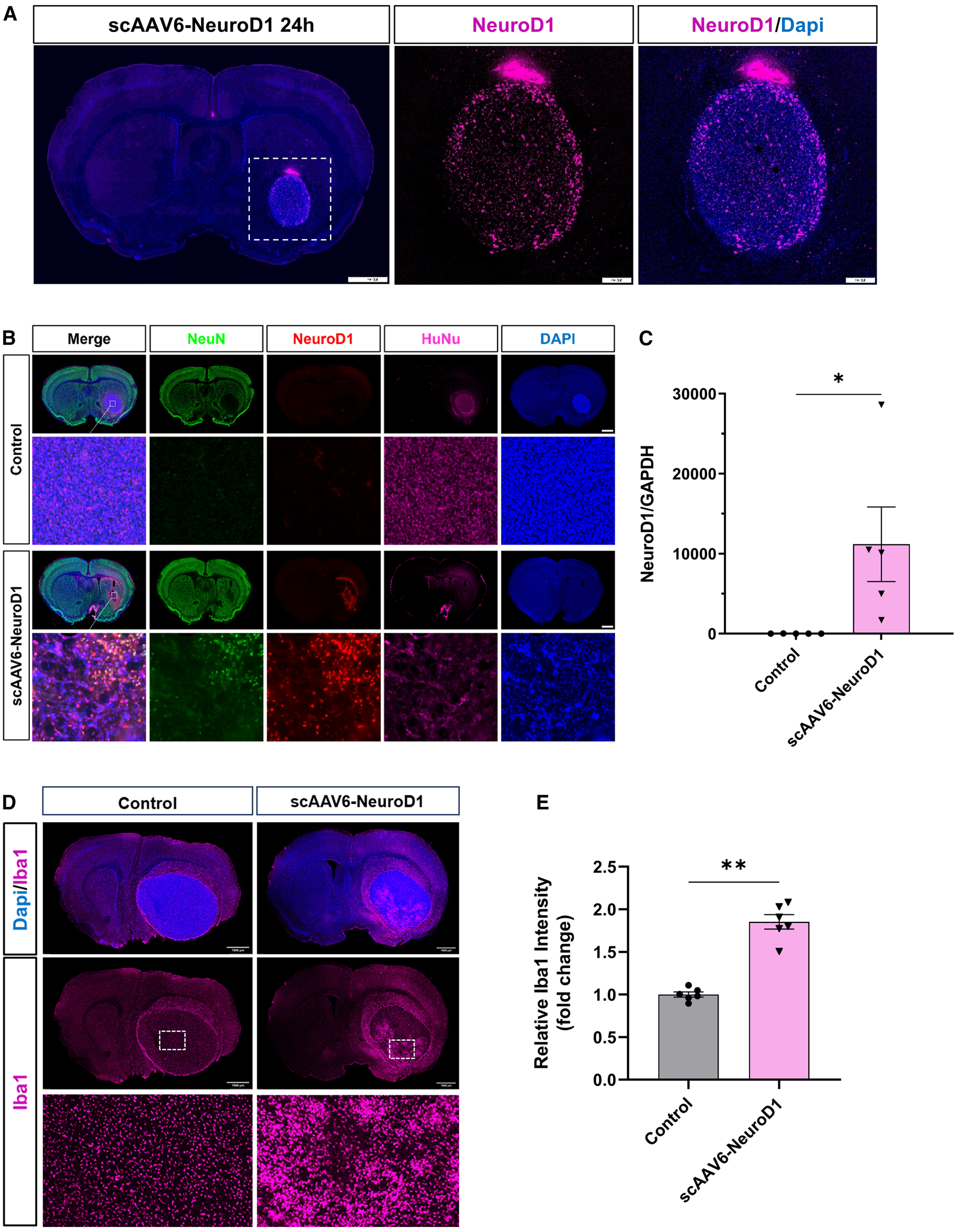

Figure 6. scAAV6-NeuroD1 induces neuronal transformation and activates tumor-associated immune response in U87 GBM orthotopic model
(A) Representative images of DAPI and NeuroD1 immunostaining from U87-MG GBM orthotopic models 24 h post-injection of scAAV6-NeuroD1. Right panels are enlarged areas from left panel. DAPI and NeuroD1 immunostaining revealed widespread and robust NeuroD1 expression within the tumor area as early as 24 h after scAAV6-NeuroD1 administration. Scale bars, 1,000 μm. (B) Immunofluorescent staining of brain sections from U87-MG GBM orthotopic models treated with scAAV6-NeuroD1 or control. The scAAV6-NeuroD1-treated group shows increased expression of NeuroD1 (red) and NeuN (green), indicating glioma-to-neuron conversion, along with positive HuNu staining

(legend continued on next page)

Further analysis revealed elevated *NEUROD1* expression in IDH mutant (MT) gliomas than IDH wild-type (WT) tumors (Figure 8E), with no gender-specific difference observed (Figure 8F). Strikingly, *NEUROD1* levels inversely correlated with tumor grade: most grade IV patients exhibit low expression of *NeuroD1* while grade II/III patients showing high expression of *NEUROD1* (Tables S1, S2, and S3). Collectively, these results establish NeuroD1 as a marker associated with lower tumor grade and improved prognosis in glioma patients.

DISCUSSIONS

The therapeutic landscape for glioblastoma (GBM) remains challenging, with conventional treatment modalities offering limited survival benefits due to tumor heterogeneity and therapy resistance. Our study pioneers a novel approach through AAV-delivered NeuroD1-mediated cellular reprogramming of glioma cells into post-mitotic neuron-like cells, potentially paving the way for therapeutic strategies for this aggressive malignancy. This therapeutic paradigm is grounded in accumulating preclinical evidence, demonstrating that lineage reprogramming can harness tumor cell plasticity to redirect malignant cells toward terminal differentiation, effectively neutralizing their tumorigenic potential while preserving surrounding tissue integrity.[38,39]

While the reprogramming of astrocytes into neurons has shown promise in regenerative medicine—particularly for neurodegenerative diseases like stroke,[11] Huntington disease,[12,40] and Alzheimer disease[10]—its application in oncology, specifically for treatment of GBM, remains largely unexplored due to concerns about tumor cell plasticity and microenvironmental interference. Our findings suggest that NeuroD1, a neurogenic transcription factor, can be repurposed to not only reprogram glial cells but also suppress GBM proliferation by converting tumor cells into neuron-like cells. This dual action of simultaneous growth inhibition and cellular identity remodeling addresses tumor complexity by targeting both proliferative capacity and malignant phenotype.

In the present study, we chose a non-replicating AAV vector primarily for its established safety profile, which is a crucial consideration for intracerebral gene therapy. The small size of AAV also favors its spread within tumors compared to larger viral vectors such as Herpes simplex virus (HSV) and adenovirus, especially as the extracellular matrix can hinder viral spread in the tumor microenvironment.[41] Moreover, AAV can be routinely produced at substantially higher titers than conventional oncolytic viruses, supporting efficient tumor transduction. AAV also exhibits low immunogenicity with no associated risk of viral encephalitis or meningitis.[42] While replicating or conditionally replicating vectors could theoretically enhance intratumoral distribution, controlling their replication within the brain tissue remains a substantial challenge, because their potential neurotoxicity raises significant safety concerns. Our choice of a non-replicating AAV serotype reflects a risk-benefit balance that prioritizes safety and translational feasibility. Future studies may explore tumor-selective promoters, engineered capsids with improved tropism, or conditionally replicating vectors, pending thorough safety validation.

We used AAV serotype 6 to construct a self-complementary AAV vector carrying NeuroD1 and evaluated its efficacy across diverse GBM models, including *in vitro* cell line cultures, patient-derived organoids, and orthotopic *in vivo* models. The therapeutic efficacy was consistently demonstrated throughout these experimental systems, underscoring its potential applicability against heterogeneous GBM subtypes. Notably, the organoid model, which better recapitulates patient tumor heterogeneity than traditional GBM cell lines, demonstrated significant upregulation of neuronal marker and reduction in tumor marker expression. Meanwhile, off-target infection of scAAV6-NeuroD1 in healthy brain tissue did not result in detectable neuronal or glial pathology (Figure S3), supporting the favorable safety profile of intracranial delivery. These findings indicate that scAAV6-NeuroD1 induces multilevel therapeutic effects, including molecular reprogramming of tumor cells, robust tumor suppression, and preservation of neurological integrity of healthy tissue, thereby supporting its translational potential as a novel gene therapy for heterogeneous GBM.

Most strikingly, scAAV6-NeuroD1 treatment in the CDX model significantly extended survival in a dose-dependent manner, a remarkable outcome given the aggressiveness of the U87-MG model known for rapid progression and therapy resistance. Furthermore, the combination of scAAV6-NeuroD1 and a frontline chemotherapeutic agent Temozolomide (TMZ) resulted in a significant survival benefit, with some animals in the combination group surviving well beyond the typical lifespan observed with either treatment alone. The synergistic effect observed suggests that reprogramming may sensitize tumor cells to chemotherapy via differentiation-induced vulnerability such as metabolic state change. Alternatively, TMZ may regulate signaling pathways such as PI3K/AKT and modulate chromatin accessibility to enhance NeuroD1-mediated neuronal conversion. Further investigations of the transcriptomic and epigenetic changes underlying this synergy are essential to fully elucidate the combined therapeutic effects. This finding underscores the potential of integrating gene therapy with other treatment modalities to enhance therapeutic efficacy.

While these results are promising, several challenges require resolution before clinical translation. First, dosing strategies optimization

(purple) marking human nuclei, compared to the control group that showed no NeuroD1 or NeuN expression. DAPI (blue) stains cell nuclei. Scale bars, 1,000 μm. (C) RT-qPCR quantification of NeuroD1 expression in the tumor area demonstrating significantly higher levels of NeuroD1 mRNA in the scAAV6-NeuroD1-treated group compared to the control. Data are presented as mean ± SEM. $^{**}p < 0.01$ by unpaired two-tailed Student's *t* test. (D) Immunofluorescent staining for Iba1, a microglia/macrophage marker, showing enhanced activation of immune cells in the tumor area of the scAAV6-NeuroD1-treated group compared to the control. The lower panels provide a higher magnification view of the tumor region. Scale bars, 1,000 μm. (E) Quantification of Iba1 immunofluorescence intensity showing a significant increase in microglia/macrophage activation in the scAAV6-NeuroD1-treated group compared to the control. Data are presented as mean ± SEM. $^{**}p < 0.01$ by unpaired two-tailed Student's *t* test.

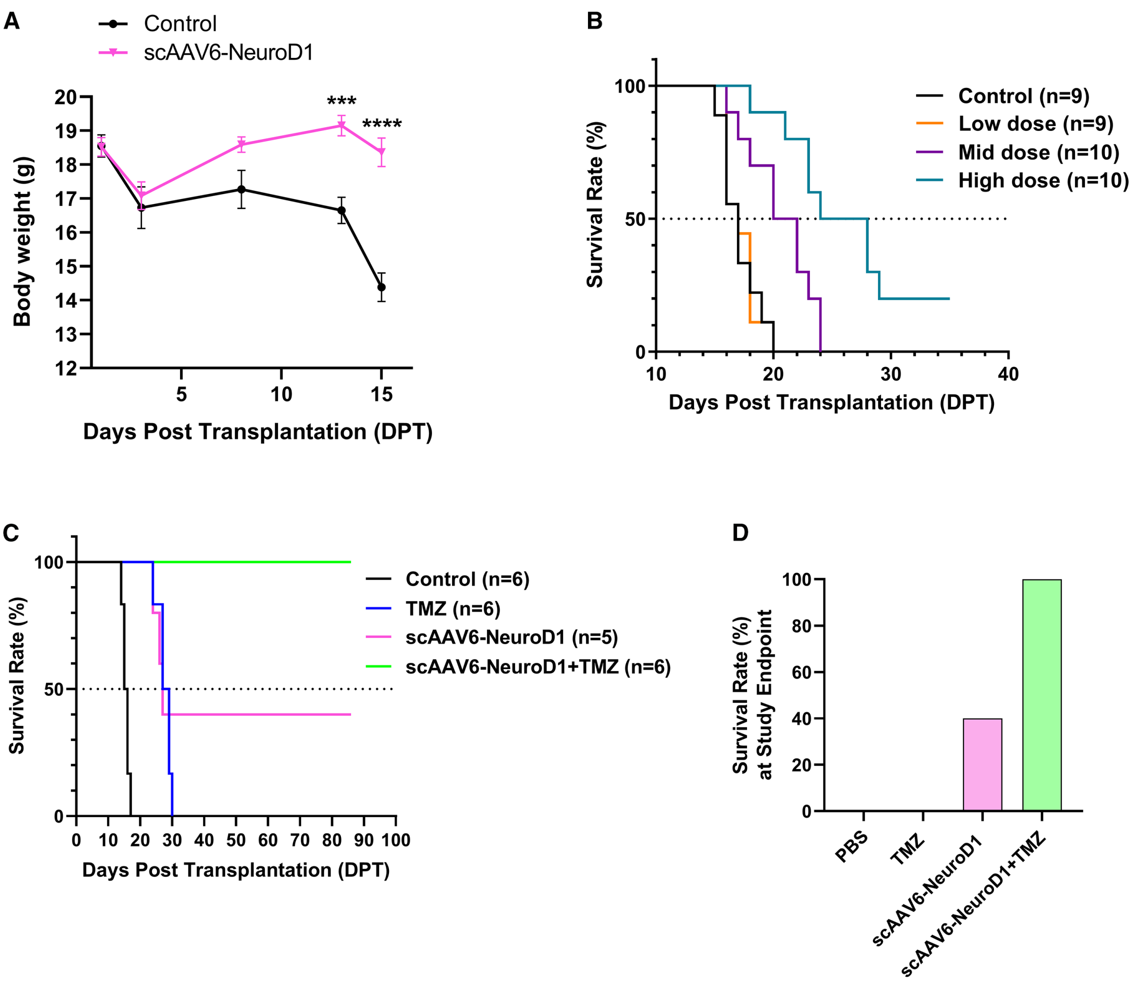

Figure 7. scAAV6-NeuroD1 delays body weight loss and prolongs survival in a U87 GBM orthotopic mouse model

(A) Body weight monitoring of mice treated with scAAV6-NeuroD1 or control showing that the scAAV6-NeuroD1-treated group maintained stable body weight until 16 days post-transplantation (DPT), whereas the control group exhibited significant weight loss starting at 13 DPT. Data are presented as mean ± SEM ($n = 6$ per group). ***$p < 0.001$, ****$p < 0.0001$ by two-way ANOVA followed by Tukey's post hoc test. (B) Kaplan-Meier survival analysis revealing that scAAV6-NeuroD1 treatment significantly extended the median survival of the mice in a dose-dependent manner. Low-dose: 6×10^{11} vg/mL; mid-dose: 2×10^{12} vg/mL; high-dose 2×10^{13} vg/mL, at volume of 4 μL. The median survival times were 17 days for both the control and low-dose groups, 22.5 days for the mid-dose group, and 26 days for the high-dose group. Data are presented as mean ± SEM (n = 9–10 per group). $p < 0.0001$ by log rank (Mantel-Cox) test. (C) Survival curves comparing the effects of scAAV6-NeuroD1 alone, TMZ (Temozolomide) alone, and their combination. The median survival times were 15.5 days for control, 27 days for scAAV6-NeuroD1 alone, and 28 days for TMZ alone, and no deaths were observed in the combination treatment group by the end of the 86-day observation period. Data are presented as mean ± SEM (n = 5–6 per group). $p < 0.0001$ by log rank (Mantel-Cox) test. (D) Survival rate at 86 DPT study point demonstrating the synergistic effect of scAAV6-NeuroD1 and TMZ, with the combination treatment group showing a 100% survival rate compared to 40% for the scAAV6-NeuroD1 monotherapy group and 0% for the TMZ monotherapy group.

must balance therapeutic efficacy and immune response to AAV capsid. Our dosing strategy in this study, based on the finding[37] that intracerebral redosing within 14 days minimizes neutralizing antibody induction, provides a viable approach to maximize the efficacy of repeated AAV administration while mitigating antiviral immunity. Further studies, particularly in non-human primate (NHP) models, will be essential to help future clinical translation. Second, comprehensive and long-term safety assessments of scAAV6-NeuroD1 are needed to evaluate risks like off-target effects in non-tumor cell population. The possibility of neural circuit disruption and seizure induction due to tumor cell conversion, while not observed in short-term murine models, warrants rigorous safety evaluation in long-term clinic-relevant models. Additionally, potential hyperexcitability of converted neurons and the consequent risks of hypoxia and angiogenesis require further evaluation. Future studies using non-human primate models will be essential for long-term tracking of AAV DNA persistence and functional assessment of converted neuron integration, providing critical safety data for clinical translation. Finally, delivery strategies must overcome the blood-brain barrier and tumor-specific targeting limitations to minimize systemic

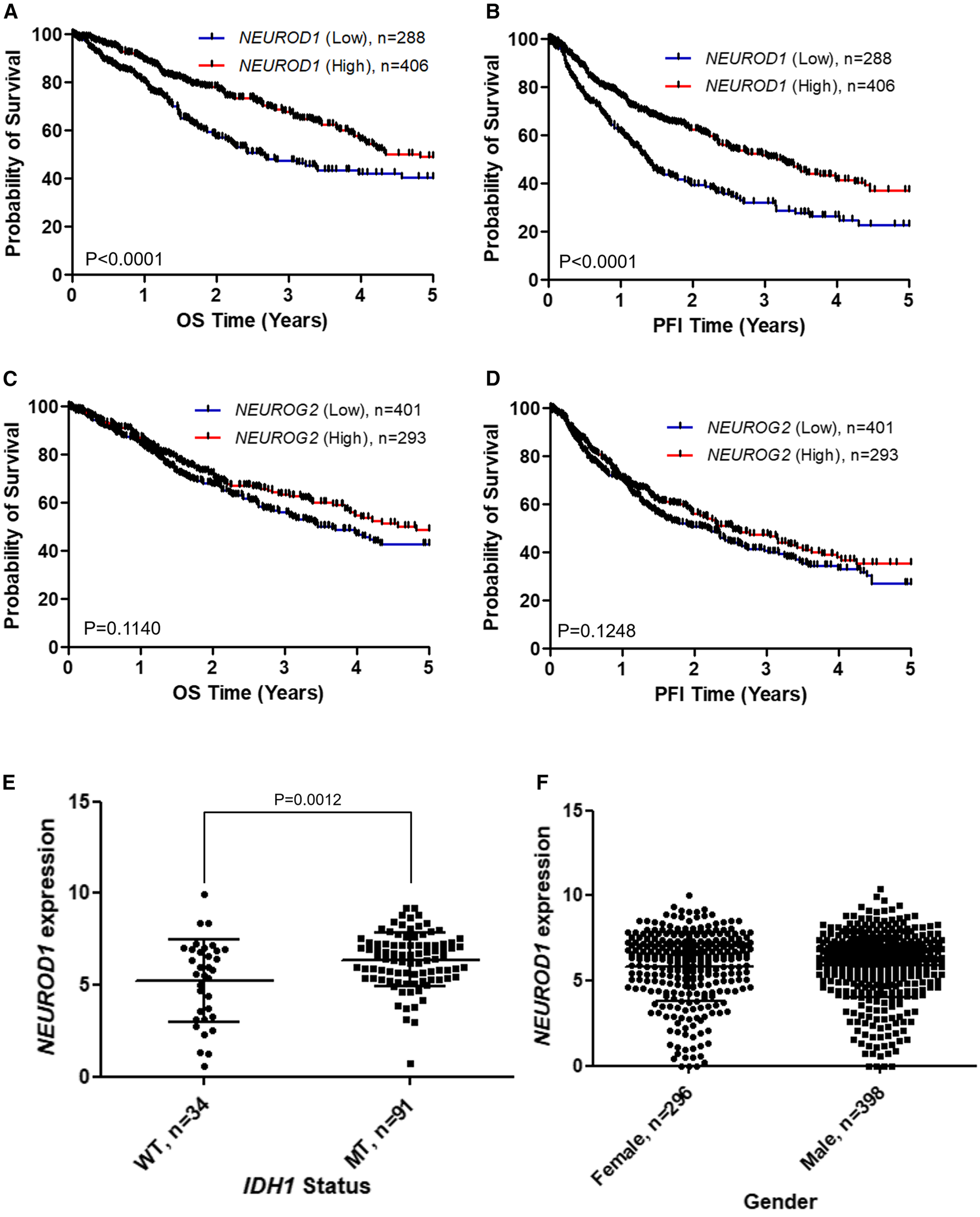

Figure 8. NeuroD1 expression inversely correlated with poor prognosis of glioma patients

(A–D) Kaplan-Meier plot of overall survival (OS) and progression-free interval (PFI) in glioma patients from TCGA lower grade glioma and glioblastoma (GBMLGG) cohort, stratified by *NEUROD1* and *NEUROG2* expression (gene expression RNAseq: IlluminaHiSeq). The *NEUROD1* and *NEUROG2* expression levels were classified into two groups: the low *NEUROD1/NEUROG2* group was relative expression value <cohort mean expression value, while the high *NEUROD1/NEUROG2* group was relative expression value >cohort mean expression value. The log rank test was used to compare differences between groups (n = 694). (E) The relative expression of *NEUROD1* in *IDH1* wild-type (WT) and *IDH1* mutation (MT) of glioma patients in TCGA GBMLGG cohort (n = 125). Data are shown as mean ± SD. p = 0.0012 by two-tailed unpaired t test. (F) Relative expression of *NEUROD1* in female and male of glioma patients within TCGA GBMLGG cohort (n = 694). Data are shown as mean ± SD. p = 0.6025 by two-tailed unpaired t test.

exposure. Although the AAV6 serotype used in this study exhibits infection preference toward glioma cells through local administration, future efforts may focus on optimizing systemic delivery methods, such as screening novel AAV serotypes with enhanced blood-brain barrier penetration capabilities or improved tumor tropism.

In summary, our study demonstrates that scAAV6-NeuroD1-mediated reprogramming represents a paradigm-shifting strategy for GBM treatment. By exploiting glioma cell plasticity, this gene therapy dual-targets proliferation and malignancy, offering a foundation for clinical translation. Future efforts should focus on refining delivery systems and validating safety, paving the way for combination therapies that integrate gene editing, immunotherapy, and precision oncology.

MATERIALS AND METHODS

AAV vector design and preparation

The following recombinant AAVs (rAAVs) were used in our study: scAAV9-CMV-NeuroD1, ssAAV6-CMVEnhancer (CE)-GFAP::NeuroD1, ssAAV9-CMVEnhancer (CE)-GFAP::NeuroD1, scAAV9-CMV::GFP, ssAAV9-GFAP::GFP, ssAAV6-GFAP::GFP and scAAV6-CMV::GFP, and scAAV6-CMV::NeuroD1). scAAV6-CMV::NeuroD1 is a self-complementary recombinant AAV (scAAV) serotype 6 vector that expresses NeuroD1 under the control of the CMV promoter, and the vector is manufactured by Packgene.

Three plasmids (GOI plasmid and two helper plasmids) will be triple transiently transfected into suspended HEK293 cells to produce replication-defective AAV6: (1) the GOI plasmid has 4,881 bps and encodes target gene for NEUROD1. The other two plasmids are (1) helper plasmid (P1001), which has 11,584 bps encoding adeno VA, adeno E4, and adeno E2A mediating AAV replication and (2) RC plasmid (ST063/P2003), which has 7,344 bps encoding Rep (required for the AAV life cycle) and AAV6 capsid protein. AAV vector copy number was determined via relative RT-qPCR quantification (QuantStudio Real-Time PCR System, Thermo Fisher Scientific). In detail, the absolute gene copy number of GFP or H19 was normalized to the absolute gene copy number of the reference gene Ptbp2 (polypyrimidine tract binding protein 2; two copies per diploid genome).

GBM cell lines and culture

Three GBM cell lines (U87-Luc, 251-Luc, and GL261-Luc) and human-derived glioma cells (BN2276, BN2338, and BN9224, provided by Crown Bioscience) were used in our study. The U87-Luc (purchased from German Collection of Microorganisms and Cell Cultures GmbH, Braunschweig) was maintained in DMEM supplemented with 10% fetal bovine serum (FBS) and 1% penicillin-streptomycin (P/S) and cultured at 37°C and 5% CO_2.

Screening of AAV serotypes and promoters

U87 cells were seeded in a 24-well plate at a density of 1×10^5 cells per well. After 4 h, the medium was discarded and replaced with 1 mL of fresh medium containing AAV6-CMV-GFP, AAV6-GFAP-GFP, AAV9-CMV-GFP, or AAV9-GFAP-GFP for 48 h. Then cells were photographed by microscopy and collected in 1.5 mL tubes and resuspended with PBS.

In vitro cell culture efficacy assay

Three GBM cell lines (U87-Luc, U251-Luc, and GL261-Luc) were seeded in 24-well plates at a density of 2×10^4 cells per well. After 4 h, medium was discarded and replaced with 1 mL of fresh medium containing 10-fold serially diluted scAAV6-NeuroD1 (multiplicity of infection MOI:10^6 to 10^3) for 48 h. Then cells were first photographed by fluorescent microscopy and collected in 1.5 mL tubes and resuspended with PBS for flow cytometry test. Cell viability was determined by the CCK-8 assay (Cell Counting Kit-8, Dojindo Laboratories) following the manufacture's manual 72 h post-infection, and the cell viability of the PBS group was used as 100%.

GBM sphere model assay

GBM cells (U87MG-Luc) were seeded on ultra-low attachment 24-well plates at a concentration of 1,000 cells/mL in DMEM supplemented with 20 ng/mL of epidermal growth factor (EGF), 10 ng/mL of basic fibroblast growth factor , and 1× B27. Fresh media was added every 3–4 days. The cultured sphere was treated with scAAV6-NeuroD1 (MOI = 10^4) on day 8 and observed under a fluorescence microscope on day 11.

GBM organoid efficacy assay

Generation and identification of glioblastoma organoids

GBM organoid model was generated based on previous published work.[36] Briefly, GBM tumor specimens diagnosed by pathologists and radiologists were collected and transported in 10 mL of cryopreserved transport medium for glioblastoma organoids.[43] Within 1 h, the tumor specimens are processed through PBS washing, mincing, red blood cell lysis, DMEM/F12 washing, and placement in 4 mL of organoid culture medium. Organoids are maintained in an orbital shaker rotating at 120 rpm at 37°C, 5% CO_2, and 90% humidity. The culture medium was replaced every 1–4 days. After approximately 2 weeks, the organoids reached a diameter of 1–2 mm for subsequent experiments.

Efficacy assay of scAAV6-NeuroD1 in GBM organoid

NLX-004 (titer of 1E13 vg/mL) was added to the organoid culture medium at MOIs of low dose (5E5), mid dose (5E6), and high dose (1.5E7). Organoids were fixed in 4% PFA at 14 days post-infection (dpi), dehydrated in 30% sucrose, followed by frozen section and immunostaining. RNA was extracted at 14 dpi for RNA sequencing analysis.

Cell immunofluorescent staining

Cells were seeded into climbing plates at a density of 2×10^5 cells per well and incubated with 1 mL of medium containing 10% FBS for 4 h. Then AAV in 1 mL PBS was added to each well for 48-h incubation. After washing with tris-buffered saline (TBS) buffer (50 mmol/L Tris-HCl, pH 7.6, and 150 mmol/L NaCl), cells were fixed with 4%

paraformaldehyde for 15 min at room temperature. Next, cells were permeabilized with 0.1% Triton X-100 TBS for 10 min and then incubated with blocking buffer containing 5% bovine serum albumin (BSA) for 30 min at 37°C. Then, the blocking buffer was discarded, and the cell samples were incubated with primary antibodies (such as 1:1,000 dilution for NeuroD1) overnight at 4°C, washed 3 times with TBS, and incubated in secondary antibodies (1:500) for 30 min at 37°C. Finally, cells were covered with anti-fluorescence quencher (Beyotime Biotechnology) containing 40,6-diamidino-2-phenylindole (DAPI) and imaged using an Olympus CLSM (FV1000).

Efficacy studies using PDX model

PDX in vitro *culture test*

The experiment involved testing two GBM-derived PDX models (BN2276 and BN9224) using a 3D culture method to determine the IC50 values of test compounds. The cells were cultured in DMEM/F12 medium supplemented with 10% FBS, Penicillin-Streptomycin, and additional growth factors. Cell viability was measured using the CellTiter-Glo assay, with Cisplatin serving as the positive control. Various instruments, including a microplate reader, CO2 incubator, and live cell counter, were utilized to perform the experiments. The IC50 values provided insights into the compound's inhibitory effects across different PDX models.

PDX in vivo *subcutaneous model test*

NOD/SCID female mice were implanted with GBM tumor tissue with diameter of around 2–3 mm subcutaneously to evaluate the efficacy of the test article. Fifty microliters of scAAV6-NeuroD1 were administered via intratumoral injection every 5 days after the tumor volume reached about 120 mm^3. Tumor growth was regularly measured, and the tumor volume was recorded to assess efficacy, while body weight monitoring helped evaluate safety.

Animal experiments

Eight-week-old BALB/c nude mice were purchased from Zheijing Vital River Laboratory Animal Technology Co., Ltd. (Zhejiang, China). All animal procedures were performed under protocols approved by the CRADL-SZ (Charles River Accelerator & Development Lab, Suzhou) Institutional Animal Care and Use Committee (IACUC). All animal experiments were performed under pathogen-free conditions. Mice were euthanized if their body weight dropped by more than 20%. Mice were randomly assigned to treatment and control groups and injected with tumor cells or rAAVs into striatum by stereotactic injection.

Efficacy studies in the U87-MG model

In the orthotopic CDX model, 30,000 U87-luc cells were transplanted into the striatum of BALB/c nude mouse brains using stereotactic intracranial injection. A total of 3×10^4 cells in 2 μL of PBS were injected at a rate of 0.5 μL/min using the following coordinates relative to bregma: AP = 0.50 mm, ML = −2.0 mm, and DV = −3.50 mm. On day 2 post-transplantation, IVIS assays were performed, and animals exhibiting a luciferase signal exceeding 1E4 p/s/cm^2/sr were allocated to either the treatment or the control group. scAAV6-NeuroD1 (1×10^{13} vg/mL) was administered intracranially into the tumor at 3, 8, and 13 DPT. A total of 4 μL was injected at a rate of 0.5 μL/min, with 2 μL delivered at two different depths using the same stereotactic coordinates. Control group received an equivalent volume of PBS. Brain tissues were harvested at 14 DPT for immunostaining, H&E staining, and RT-qPCR to assess the efficacy and mechanism of scAAV6-NeuroD1. For survival analysis, 40 animals (20 per group) were monitored until endpoint criteria were met, defined as a 20% loss in body weight from peak weight or the determination of the survival endpoint by a veterinarian. For TMZ combination experiment, scAAV6-NeuroD1 treatment was administered as above, while three doses of TMZ (5 mg/kg) were given intraperitoneally on days 7, 8, and 9 post-tumor establishment for the TMZ-treated or combination treatment groups.

Immunofluorescence and H&E staining for brain tissues

At the endpoint of treatment, the mice were euthanized. The brain samples were harvested and fixed with 4% paraformaldehyde. All samples were embedded in O.C.T Compound (Tissue-Tek) and sectioned into slices at a thickness of 40 μm by RWD FS800 Cryostats. Sample sections were then washed with PBS, and the samples were incubated in blocking buffer (5% BSA and 5% normal goat serum) for 60–120 min. For immunofluorescence staining, samples were incubated with different primary antibodies overnight at 4°C, washed with PBS, and incubated with fluorescently labeled secondary antibodies (1:1,000) for 60–120 min at room temperature. For H&E staining, sections were stained using an assay kit (Vector Laboratories) according to the manufacturers' protocols. Last, the slides were imaged and analyzed using a Research Slide Scanner microscope (Olympus VS200).

Efficacy studies in the GL261 model

Male C57BL/6 mice (4 weeks old) were purchased from Vital River Laboratories (Beijing, China) and maintained under specific pathogen-free conditions. The animals were randomly assigned to control and experimental groups. An orthotopic glioma model was established by stereotactic intracranial implantation of 5×10^5 luciferase-expressing GL261 cells (GeneChem, China) into the striatum (AP = 0.50 mm, ML = −2.0 mm, and DV = −3.50 mm relative to bregma). The treatment group received scAAV6-NeuroD1 at the original tumor site on days 6, 11, and 16 post-implantation, while controls received PBS on the same schedule. Tumor progression was monitored using an IVIS imaging system (Caliper Life Sciences, USA) 24 h before treatment (days 5, 10, and 15) following intraperitoneal injection of D-luciferin (Solarbio, China). Body weight and health status were recorded throughout the study. Brain tissues were collected upon mortality, fixed in 4% paraformaldehyde, and processed for immunohistochemistry following protocol above.

Flow cytometry

Mice were transcardially perfused with ice-cold PBS. The whole brain was collected and mechanically dissociated through a 70-μm

cell strainer. The homogenate was digested with a cocktail of Collagenase D (1 mg/mL) and DNase I (0.1 mg/mL) in HBSS at 37°C for 30 min with agitation. The cell suspension was centrifuged through a 30% Percoll gradient to enrich leukocytes and remove myelin debris. After the isolated cells were washed and counted, Fc receptors were blocked using an anti-CD16/32 antibody. Cells were stained with a fluorochrome-conjugated antibody cocktail against CD4 and CD8 for 30 min at 4°C in the dark.

For flow cytometric analysis, single cells were gated by excluding doublets based on FSC-H versus FSC-A parameters. Viable lymphocytes were identified from the single-cell population based on forward and side scatter properties (FSC-A/SSC-A), effectively excluding cell debris and dead cells. Within the viable lymphocyte gate, the immune microenvironment was assessed by analyzing the expression of CD4 and CD8 to identify helper and cytotoxic T cell subsets, respectively. Data were acquired on a flow cytometer and analyzed using FlowJo software.

RNA isolation reverse transcriptase-quantitative PCR

Brain tissues were harvested, and tumor sections were collected for mRNA analysis. RNA and DNA were extracted using the AllPrep DNA/RNA Mini Kit (Qiagen). Reverse transcription was performed using the Transcriptor First Strand cDNA Synthesis Kit (Roche), and RT-qPCR was then performed using the QuantiNova SYBR Green PCR Kit on the QuantStudio 6 Pro Real-Time PCR System.

Experimental observation and data collection

After inoculation of tumor cells, animals were routinely monitored for general behavior, activity levels, food and water intake, weight changes, and physical condition. Symptoms observed were recorded, and mice were weighed two or three times weekly, with frequency increasing as they approached the survival endpoint.

Statistics

Statistical analyses were conducted using GraphPad Prism Software v.9.3.1. Mann-Whitney tests were used to analyze RT-PCR data significance between experimental groups, with a one- or two-tailed p value calculated accordingly. Survival analysis was performed using Kaplan-Meier plots, with significance determined by the log rank test (Mantel-Cox).

Clinical data analysis

Clinical data analysis was performed using publicly available datasets from the TCGA lower grade glioma and glioblastoma (GBMLGG) cohort (UCSC Xena) and three cohorts from the Chinese Glioma Genome Atlas (CGGA). Kaplan-Meier survival analysis was conducted to assess the correlation between NEUROD1 and NEUROG2 expression and OS and PFI, with statistical significance determined by the log rank test. Expression differences between groups (e.g., gender, IDH mutant vs. wild type, and different WHO grades) were analyzed using the two-tailed unpaired t test. All analyses were conducted using GraphPad Prism (v.9.3.1), with a p value <0.05 considered significant.

DATA AND CODE AVAILABILITY

The data that support the findings of this study are available from the corresponding author upon reasonable request.

ACKNOWLEDGMENTS

This study is supported by fundings from NeuExcell Therapeutics, National Natural Science Foundation of China Project Nos. 82173279 and 82472981(to Y.H.); National Science and Technology Resource Sharing Service Platform Project No. YCZYPT [2020]06-1 (to Y.H.); Suzhou Medical and Health Innovation Project No. CXYJ2024A05 (to Y.H.); and Suzhou Science and Technology Plan Project No. SZM2023019 (to X.L.).

AUTHOR CONTRIBUTIONS

Y.C., S.J., Y.H., and G.C. conceptualized the research. S.J., M.L., M.C., T.-C.K., K.Z., Z.J., J. Xie, and H.Z. performed all experiments with assistance of L.P., S.C., A.S.C., and Y.C. M.C., J. Xu, Q.W., and X.L. provided suggestions during the research and the writing. Y.C. and G.C. wrote the manuscript. All authors reviewed, edited, and approved the final version of the manuscript.

DECLARATION OF INTERESTS

G.C. is a co-founder of NeuExcell Therapeutics. Y.C., S.J., M.L., M.C., T.-C.K., K.Z., L.P., M.C., S.C., A.S.C., J. Xu, and S.J. are/were employees of NeuExcell Therapeutics. The authors declare that a patent application for this work has been filed and is currently under review.

DECLARATION OF GENERATIVE AI AND AI-ASSISTED TECHNOLOGIES IN THE WRITING PROCESS

During the preparation of this work, the author(s) used DeepSeek in order to improve readability and language during writing. After using this tool/service, the author(s) reviewed and edited the content as needed and take(s) full responsibility for the content of the publication.

SUPPLEMENTAL INFORMATION

Supplemental information can be found online at https://doi.org/10.1016/j.omton.2026.201205.

REFERENCES

1. Wen, P.Y., Weller, M., Lee, E.Q., Alexander, B.M., Barnholtz-Sloan, J.S., Barthel, F.P., Batchelor, T.T., Bindra, R.S., Chang, S.M., Chiocca, E.A., et al. (2020). Glioblastoma in adults: a Society for Neuro-Oncology (SNO) and European Society of Neuro-Oncology (EANO) consensus review on current management and future directions. Neuro Oncol. *22*, 1073–1113. https://doi.org/10.1093/neuonc/noaa106.
2. Tan, A.C., Ashley, D.M., López, G.Y., Malinzak, M., Friedman, H.S., and Khasraw, M. (2020). Management of glioblastoma: State of the art and future directions. CA Cancer J. Clin. *70*, 299–312. https://doi.org/10.3322/caac.21613.
3. Louis, D.N., Perry, A., Wesseling, P., Brat, D.J., Cree, I.A., Figarella-Branger, D., Hawkins, C., Ng, H.K., Pfister, S.M., Reifenberger, G., et al. (2021). The 2021 WHO Classification of Tumors of the Central Nervous System: a summary. Neuro Oncol. *23*, 1231–1251. https://doi.org/10.1093/neuonc/noab106.
4. Stupp, R., Mason, W.P., van den Bent, M.J., Weller, M., Fisher, B., Taphoorn, M.J.B., Belanger, K., Brandes, A.A., Marosi, C., Bogdahn, U., et al. (2005). Radiotherapy plus concomitant and adjuvant temozolomide for glioblastoma. N. Engl. J. Med. *352*, 987–996. https://doi.org/10.1056/NEJMoa043330.
5. Gilbert, M.R., Dignam, J.J., Armstrong, T.S., Wefel, J.S., Blumenthal, D.T., Vogelbaum, M.A., Colman, H., Chakravarti, A., Pugh, S., Won, M., et al. (2014). A randomized trial of bevacizumab for newly diagnosed glioblastoma. N. Engl. J. Med. *370*, 699–708. https://doi.org/10.1056/NEJMoa1308573.
6. Weller, M., van den Bent, M., Preusser, M., Le Rhun, E., Tonn, J.C., Minniti, G., Bendszus, M., Balana, C., Chinot, O., Dirven, L., et al. (2021). EANO guidelines on the diagnosis and treatment of diffuse gliomas of adulthood. Nat. Rev. Clin. Oncol. *18*, 170–186. https://doi.org/10.1038/s41571-020-00447-z.

7. Gao, Z., Ure, K., Ables, J.L., Lagace, D.C., Nave, K.A., Goebbels, S., Eisch, A.J., and Hsieh, J. (2009). Neurod1 is essential for the survival and maturation of adult-born neurons. Nat. Neurosci. *12*, 1090–1092. https://doi.org/10.1038/nn.2385.

8. Matsuda-Ito, K., Matsuda, T., and Nakashima, K. (2022). Expression level of the reprogramming factor NeuroD1 is critical for neuronal conversion efficiency from different cell types. Sci. Rep. *12*, 17980. https://doi.org/10.1038/s41598-022-22802-z.

9. Li, H., and Chen, G. (2016). In Vivo Reprogramming for CNS Repair: Regenerating Neurons from Endogenous Glial Cells. Neuron *91*, 728–738. https://doi.org/10.1016/j.neuron.2016.08.004.

10. Wu, Z., Xu, L., Xie, Y., Sambangi, A., Swaminathan, S., Pei, Z., Ji, W., Li, Z., Guo, Y., Li, Z., and Chen, G. (2025). Brain-Wide Neuroregenerative Gene Therapy Improves Cognition in a Mouse Model of Alzheimer's Disease. Adv. Sci. *12*, e2410080. https://doi.org/10.1002/advs.202410080.

11. Chen, Y.-C., Ma, N.-X., Pei, Z.-F., Wu, Z., Do-Monte, F.H., Keefe, S., Yellin, E., Chen, M.S., Yin, J.-C., Lee, G., et al. (2020). A NeuroD1 AAV-Based Gene Therapy for Functional Brain Repair after Ischemic Injury through In Vivo Astrocyte-to-Neuron Conversion. Mol. Ther. *28*, 217–234. https://doi.org/10.1016/j.ymthe.2019.09.003.

12. Wu, Z., Parry, M., Hou, X.-Y., Liu, M.-H., Wang, H., Cain, R., Pei, Z.-F., Chen, Y.-C., Guo, Z.-Y., Abhijeet, S., and Chen, G. (2020). Gene therapy conversion of striatal astrocytes into GABAergic neurons in mouse models of Huntington's disease. Nat. Commun. *11*, 1105. https://doi.org/10.1038/s41467-020-14855-3.

13. Zheng, J., Li, T., Qi, S., Qin, B., Yu, J., and Chen, G. (2022). Neuroregenerative gene therapy to treat temporal lobe epilepsy in a rat model. Prog. Neurobiol. *208*, 102198. https://doi.org/10.1016/j.pneurobio.2021.102198.

14. Puls, B., Ding, Y., Zhang, F., Pan, M., Lei, Z., Pei, Z., Jiang, M., Bai, Y., Forsyth, C., Metzger, M., et al. (2020). Regeneration of Functional Neurons After Spinal Cord Injury via in situ NeuroD1-Mediated Astrocyte-to-Neuron Conversion. Front. Cell Dev. Biol. *8*, 591883. https://doi.org/10.3389/fcell.2020.591883.

15. Wang, H., Zhao, P., Zhang, Y., Chen, Z., Bao, H., Qian, W., Wu, J., Xing, Z., Hu, X., Jin, K., et al. (2023). NeuroD4 converts glioblastoma cells into neuron-like cells through the SLC7A11-GSH-GPX4 antioxidant axis. Cell Death Discov. *9*, 297. https://doi.org/10.1038/s41420-023-01595-8.

16. Wang, X., Pei, Z., Hossain, A., Bai, Y., and Chen, G. (2021). Transcription factor-based gene therapy to treat glioblastoma through direct neuronal conversion. Cancer Biol. Med. *18*, 860–874. https://doi.org/10.20892/j.issn.2095-3941.2020.0499.

17. Jiang, M.Q., Yu, S.P., Estaba, T., Choi, E., Berglund, K., Gu, X., and Wei, L. (2024). Reprogramming Glioblastoma Cells into Non-Cancerous Neuronal Cells as a Novel Anti-Cancer Strategy. Cells *13*, 897. https://doi.org/10.3390/cells13110897.

18. Zhao, J., He, H., Zhou, K., Ren, Y., Shi, Z., Wu, Z., Wang, Y., Lu, Y., Jiao, J., and Jiao, J. (2012). Neuronal Transcription Factors Induce Conversion of Human Glioma Cells to Neurons and Inhibit Tumorigenesis. PLoS One *7*, e41506. https://doi.org/10.1371/journal.pone.0041506.

19. Su, Z., Zang, T., Liu, M.L., Wang, L.L., Niu, W., and Zhang, C.L. (2014). Reprogramming the fate of human glioma cells to impede brain tumor development. Cell Death Dis. *5*, e1463. https://doi.org/10.1038/cddis.2014.425.

20. Cheng, X., Tan, Z., Huang, X., Yuan, Y., Qin, S., Gu, Y., Wang, D., He, C., and Su, Z. (2019). Inhibition of Glioma Development by ASCL1-Mediated Direct Neuronal Reprogramming. Cells *8*, 571. https://doi.org/10.3390/cells8060571.

21. Liu, H., Xia, J., Wang, T., Li, W., Song, Y., and Tan, G. (2019). Differentiation of human glioblastoma U87 cells into cholinergic neuron. Neurosci. Lett. *704*, 1–7. https://doi.org/10.1016/j.neulet.2019.03.049.

22. Lee, C., Robinson, M., and Willerth, S.M. (2018). Direct Reprogramming of Glioblastoma Cells into Neurons Using Small Molecules. ACS Chem. Neurosci. *9*, 3175–3185. https://doi.org/10.1021/acschemneuro.8b00365.

23. Gao, L., Huang, S., Zhang, H., Hua, W., Xin, S., Cheng, L., Guan, W., Yu, Y., Mao, Y., and Pei, G. (2019). Suppression of glioblastoma by a drug cocktail reprogramming tumor cells into neuronal like cells. Sci. Rep. *9*, 3462. https://doi.org/10.1038/s41598-019-39852-5.

24. Li, C., and Samulski, R.J. (2020). Engineering adeno-associated virus vectors for gene therapy. Nat. Rev. Genet. *21*, 255–272. https://doi.org/10.1038/s41576-019-0205-4.

25. Gao, J., Gunasekar, S., Xia, Z.J., Shalin, K., Jiang, C., Chen, H., Lee, D., Lee, S., Pisal, N.D., Luo, J.N., et al. (2024). Gene therapy for CNS disorders: modalities, delivery and translational challenges. Nat. Rev. Neurosci. *25*, 553–572. https://doi.org/10.1038/s41583-024-00829-7.

26. Wang, D., Tai, P.W.L., and Gao, G. (2019). Adeno-associated virus vector as a platform for gene therapy delivery. Nat. Rev. Drug Discov. *18*, 358–378. https://doi.org/10.1038/s41573-019-0012-9.

27. Santiago-Ortiz, J.L., and Schaffer, D.V. (2016). Adeno-associated virus (AAV) vectors in cancer gene therapy. J. Control. Release *240*, 287–301. https://doi.org/10.1016/j.jconrel.2016.01.001.

28. Luo, J., Luo, Y., Sun, J., Zhou, Y., Zhang, Y., and Yang, X. (2015). Adeno-associated virus-mediated cancer gene therapy: current status. Cancer Lett. *356*, 347–356. https://doi.org/10.1016/j.canlet.2014.10.045.

29. Maguire, C.A., Gianni, D., Meijer, D.H., Shaket, L.A., Wakimoto, H., Rabkin, S.D., Gao, G., and Sena-Esteves, M. (2010). Directed evolution of adeno-associated virus for glioma cell transduction. J. Neuro Oncol. *96*, 337–347. https://doi.org/10.1007/s11060-009-9972-7.

30. Harding, T.C., Lalani, A.S., Roberts, B.N., Yendluri, S., Luan, B., Koprivnikar, K.E., Gonzalez-Edick, M., Huan-Tu, G., Musterer, R., VanRoey, M.J., et al. (2006). AAV serotype 8-mediated gene delivery of a soluble VEGF receptor to the CNS for the treatment of glioblastoma. Mol. Ther. *13*, 956–966. https://doi.org/10.1016/j.ymthe.2006.02.004.

31. Hacker, U.T., Bentler, M., Kaniowska, D., Morgan, M., and Büning, H. (2020). Towards Clinical Implementation of Adeno-Associated Virus (AAV) Vectors for Cancer Gene Therapy: Current Status and Future Perspectives. Cancers (Basel) *12*, 1889. https://doi.org/10.3390/cancers12071889.

32. Huszthy, P.C., Svendsen, A., Wilson, J.M., Kotin, R.M., Lønning, P.E., Bjerkvig, R., and Hoover, F. (2005). Widespread dispersion of adeno-associated virus serotype 1 and adeno-associated virus serotype 6 vectors in the rat central nervous system and in human glioblastoma multiforme xenografts. Hum. Gene Ther. *16*, 381–392. https://doi.org/10.1089/hum.2005.16.381.

33. von Roemeling, C.A., Patel, J.A., Carpenter, S.L., Yegorov, O., Yang, C., Bhatia, A., Doonan, B.P., Russell, R., Trivedi, V.S., Klippel, K., et al. (2024). Adeno-associated virus delivered CXCL9 sensitizes glioblastoma to anti-PD-1 immune checkpoint blockade. Nat. Commun. *15*, 5871. https://doi.org/10.1038/s41467-024-49989-1.

34. Jacob, F., Salinas, R.D., Zhang, D.Y., Nguyen, P.T.T., Schnoll, J.G., Wong, S.Z.H., Thokala, R., Sheikh, S., Saxena, D., Prokop, S., et al. (2020). A Patient-Derived Glioblastoma Organoid Model and Biobank Recapitulates Inter- and Intra-tumoral Heterogeneity. Cell *180*, 188–204.e22. https://doi.org/10.1016/j.cell.2019.11.036.

35. Hubert, C.G., Rivera, M., Spangler, L.C., Wu, Q., Mack, S.C., Prager, B.C., Couce, M., McLendon, R.E., Sloan, A.E., and Rich, J.N. (2016). A Three-Dimensional Organoid Culture System Derived from Human Glioblastomas Recapitulates the Hypoxic Gradients and Cancer Stem Cell Heterogeneity of Tumors Found In Vivo. Cancer Res. *76*, 2465–2477. https://doi.org/10.1158/0008-5472.Can-15-2402.

36. Zhang, Y., Shao, Y., Li, Y., Li, X., Zhang, X., E, Q., Wang, W., Jiang, Z., Gan, W., and Huang, Y. (2024). The generation of glioma organoids and the comparison of two culture methods. Cancer Med. *13*, e7081. https://doi.org/10.1002/cam4.7081.

37. Xu, Y., Bai, X., Lin, J., Lu, K., Weng, S., Wu, Y., Liu, S., Li, H., Wu, Z., Chen, G., and Li, W. (2025). Intracranial AAV administration dose-dependently recruits B cells to inhibit the AAV redosing. Mol. Ther. Methods Clin. Dev. *33*, 101420. https://doi.org/10.1016/j.omtm.2025.101420.

38. Ascic, E., Åkerström, F., Sreekumar Nair, M., Rosa, A., Kurochkin, I., Zimmermannova, O., Catena, X., Rotankova, N., Veser, C., Rudnik, M., et al. (2024). In vivo dendritic cell reprogramming for cancer immunotherapy. Science *386*, eadn9083. https://doi.org/10.1126/science.adn9083.

39. Gong, L., Yan, Q., Zhang, Y., Fang, X., Liu, B., and Guan, X. (2019). Cancer cell reprogramming: a promising therapy converting malignancy to benignity. Cancer Commun. *39*, 48. https://doi.org/10.1186/s40880-019-0393-5.

40. Wu, Z., Parry, M., Hou, X.Y., Liu, M.H., Wang, H., Cain, R., Pei, Z.F., Chen, Y.C., Guo, Z.Y., Abhijeet, S., and Chen, G. (2020). Gene therapy conversion of striatal astrocytes into GABAergic neurons in mouse models of Huntington's disease. Nat. Commun. *11*, 1105. https://doi.org/10.1038/s41467-020-14855-3.

41. Henke, E., Nandigama, R., and Ergün, S. (2019). Extracellular Matrix in the Tumor Microenvironment and Its Impact on Cancer Therapy. Front. Mol. Biosci. *6*, 160. https://doi.org/10.3389/fmolb.2019.00160.
42. Ungerechts, G., Bossow, S., Leuchs, B., Holm, P.S., Rommelaere, J., Coffey, M., Coffin, R., Bell, J., and Nettelbeck, D.M. (2016). Moving oncolytic viruses into the clinic: clinical-grade production, purification, and characterization of diverse oncolytic viruses. Mol. Ther. Methods Clin. Dev. *3*, 16018. https://doi.org/10.1038/mtm.2016.18.
43. Jacob, F., Ming, G.L., and Song, H. (2020). Generation and biobanking of patient-derived glioblastoma organoids and their application in CAR T cell testing. Nat. Protoc. *15*, 4000–4033. https://doi.org/10.1038/s41596-020-0402-9.

OMTON, Volume *34*

Supplemental information

NeuroD1 gene therapy inhibits glioma growth and extends life span through *in vivo* reprogramming approach

Yuchen Chen, Zuoyu Jiang, Sen Jin, Meng Liu, Ming Chen, Tsang-Chih Kuo, Kai Zhou, Liting Pu, Ming Chen, Shiyuan Chen, Xuetao Li, Adalia S. Chen, Jingmu Xie, Huitao Zhang, Qingsong Wang, Jie Xu, Jian Sheng, Yulun Huang, and Gong Chen

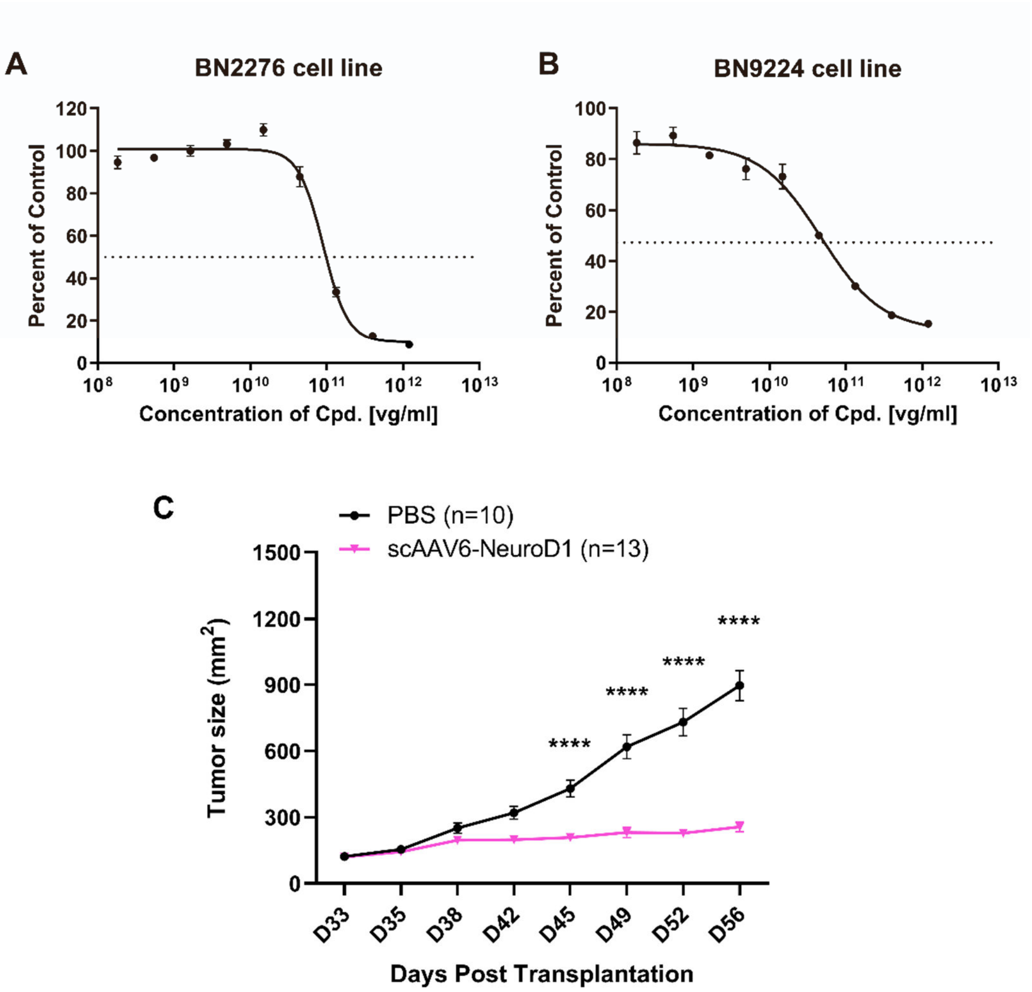

Figure S1. In Vitro and In Vivo Efficacy of scAAV6-NeuroD1 in PDX Models of Glioblastoma

(A-B) Dose-response curves illustrating the effect of scAAV6-NeuroD1 on cell viability in patient-derived glioblastoma cell lines BN2276 (A) and BN9224 (B). Cells were treated with increasing concentrations of scAAV6-NeuroD1, and viability was assessed using a CCK-8 assay. The IC50 values (dash line) were approximately 9.8×10^{11} vg/mL for BN2276 and 4.6×10^{10} vg/mL for BN9224. Data are presented as mean ± SEM (n=3 per group).

(C) Tumor growth curves in subcutaneous BN2276 glioblastoma PDX model bearing BN2276 treated with scAAV6-NeuroD1 or PBS (control). Tumor size was measured

at regular intervals post-transplantation. scAAV6-NeuroD1 treatment significantly inhibited tumor growth compared to the control group, with a marked reduction in tumor size observed by Day 56 post-transplantation. Data are presented as mean ± SEM (n=8-10 per group). ****$p<0.0001$ by two-way ANOVA followed by Tukey's post hoc test.

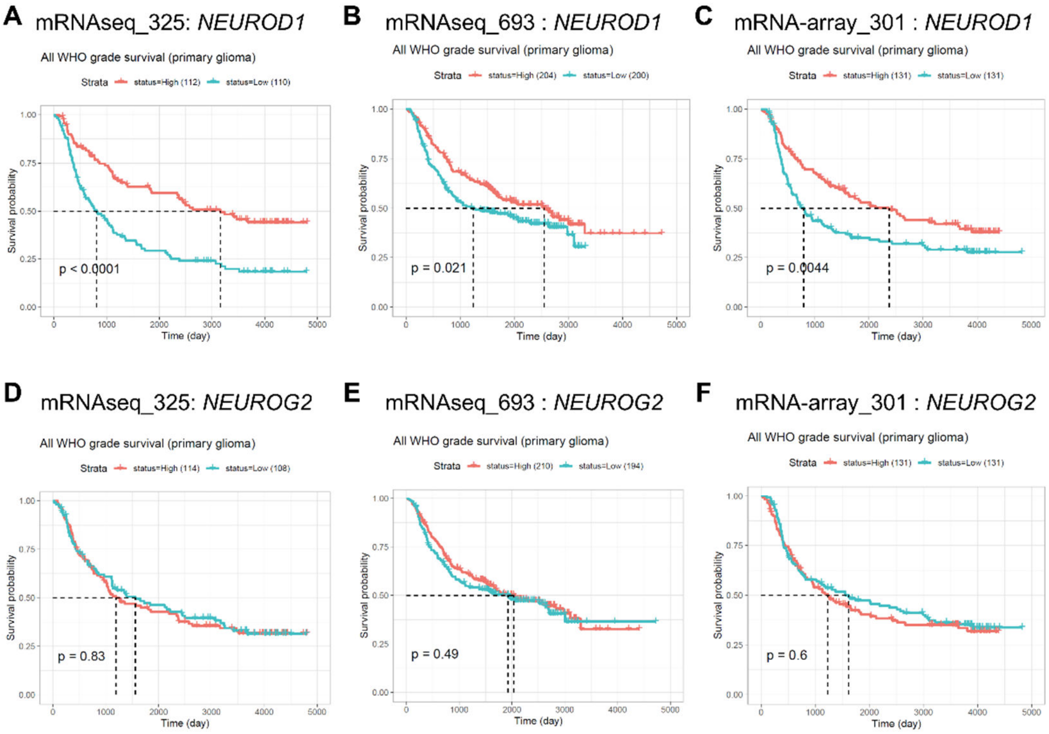

Figure S2. NeuroD1 but not Neurog2 expression positively correlated with survival in primary glioma from three CGGA cohorts.

Overall survival data of *NEUROD1* and *NEUROG2* in glioma patients from three CGGA cohorts (mRNAseq_325, mRNAseq_693 and mRNA-array_301) (**A-F**). The *NEUROD1* and *NEUROG2* expression levels were classified into low and high groups by CGGA website. Cohort: mRNAseq_325, n=222; cohort: mRNAseq_693, n=404; cohort: mRNA-array_301, n=262.

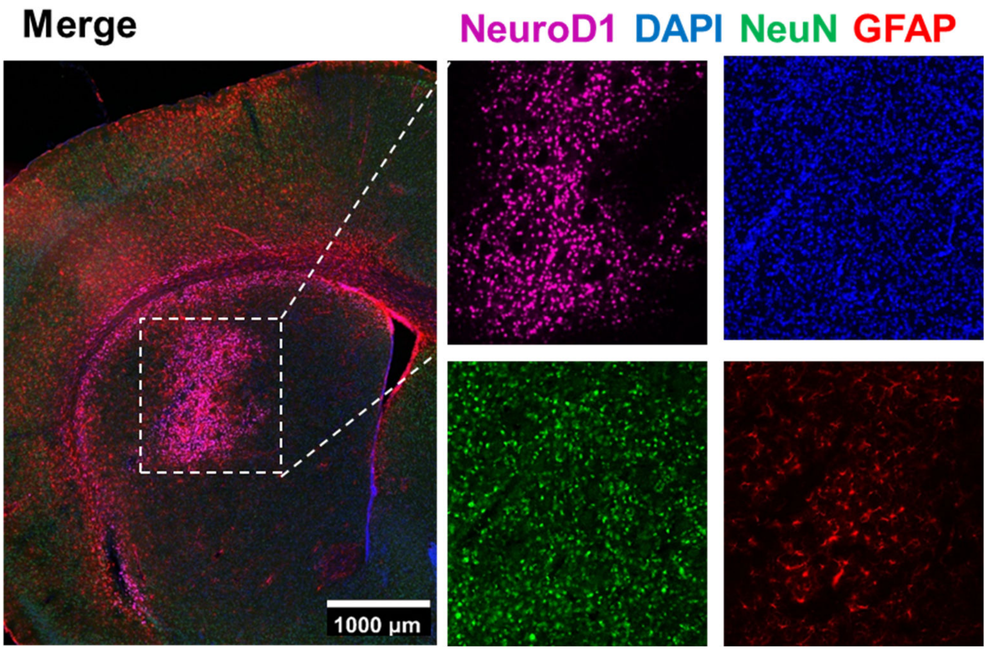

Figure S3. Transduction of scAAV6-NeuroD1 in healthy mouse brain.

Distribution of scAAV6-NeuroD1 and high magnification view of transduction of scAAV6-NeuroD1 in healthy mouse brain 3 days post-intracranial injection, showing NeuroD1 expression (magenta) in striatal areas without evident tissue disruption. Neuronal marker NeuN and DAPI marker display normal morphology and organization, with GFAP expression indicating no significant reactivity of astrocytes, indicating no significant pathological changes resulting from off-target AAV expression. Scale bar = 1000 µm.

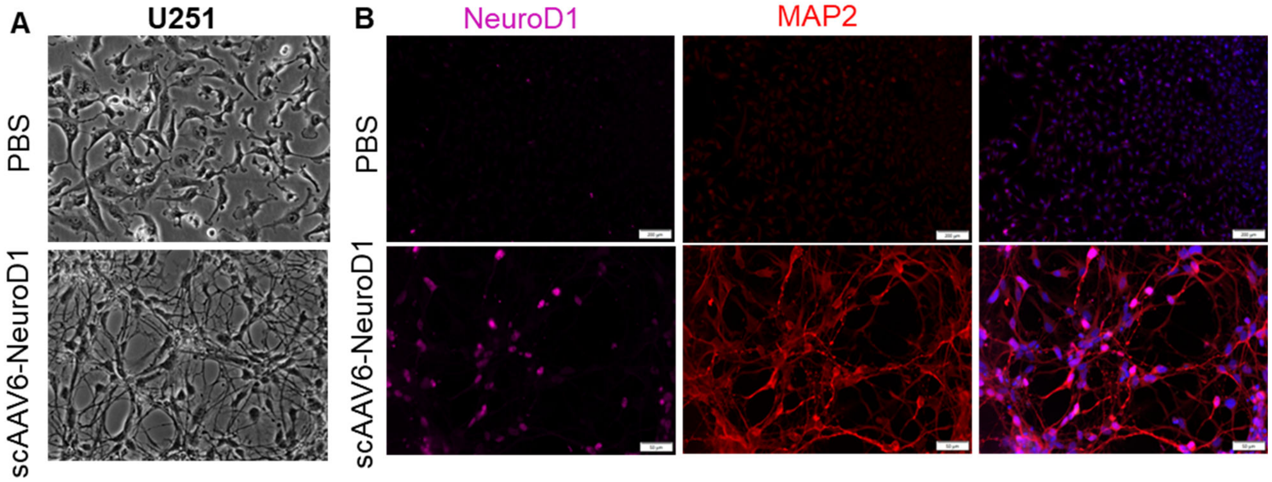

Figure S4. scAAV6-NeuroD1 induces neuronal conversion in U251 glioma cells in vitro.

(A) Phase-contrast images showing morphological changes in U251 cells following scAAV6-NeuroD1 transduction. scAAV6-NeuroD1-treated cells (lower panel) display neurite outgrowth and adopt a neuronal-like morphology at 14 days post infection.

(B) Immunofluorescence analysis of neuronal marker expression. scAAV6-NeuroD1-treated cells (bottom row) exhibit strong NeuroD1 (purple) and MAP2 (red) expression, confirming neuronal reprogramming. Nuclei are counterstained with DAPI (blue). Scale bar = 50 µm.

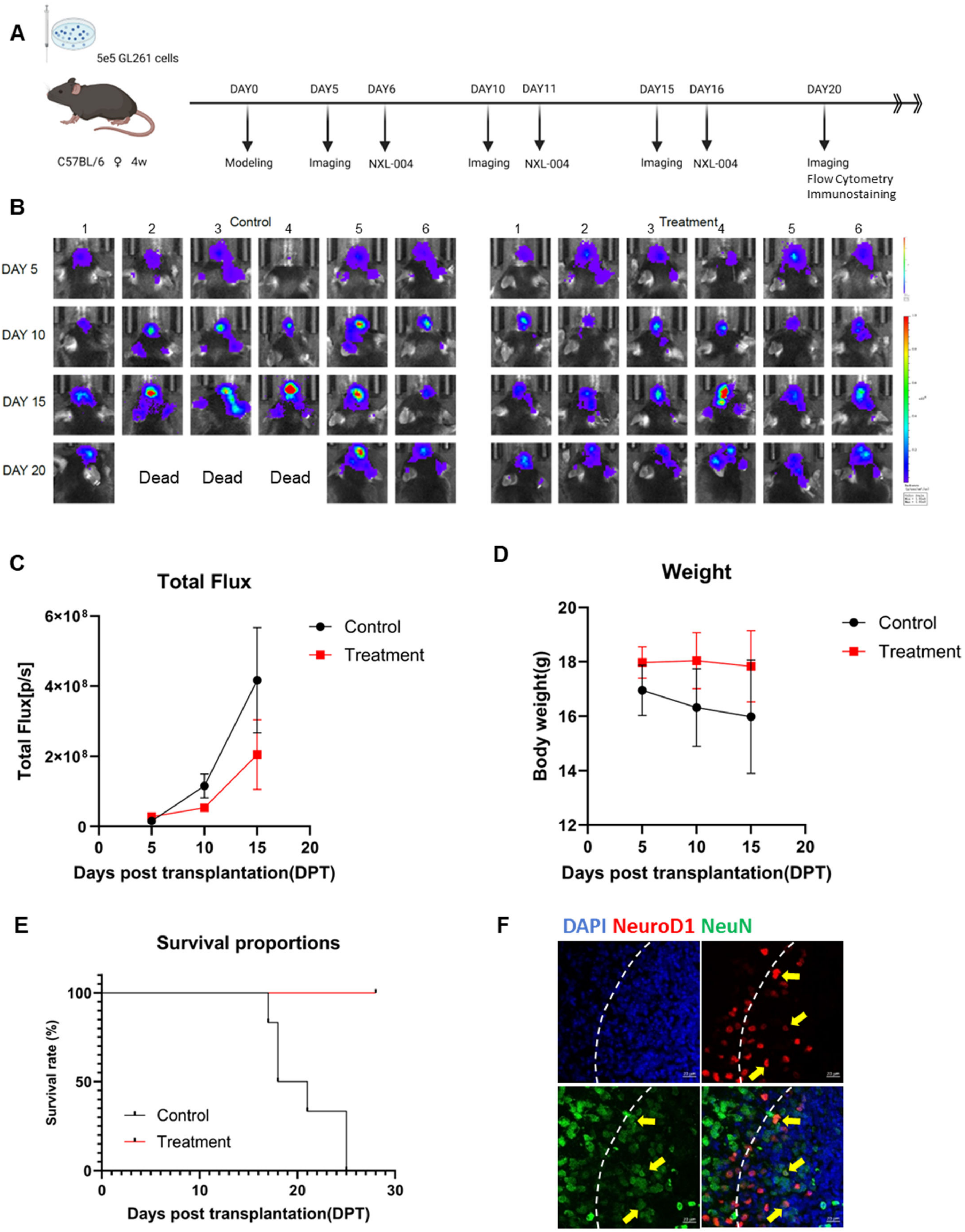
A
5e5 GL261 cells
C57BL/6 ♀ 4w
DAY0
DAY5
DAY6
DAY10
DAY11
DAY15
DAY16
DAY20
Modeling
Imaging
NXL-004
Imaging
NXL-004
Imaging
NXL-004
Imaging
Flow Cytometry
Immunostaining
B
Control
Treatment
DAY 5
DAY 10
DAY 15
DAY 20
Dead
Dead
Dead
C
Total Flux
Total Flux[p/s]
Days post transplantation(DPT)
Control
Treatment
D
Weight
Body weight(g)
Days post transplantation(DPT)
Control
Treatment
E
Survival proportions
Survival rate (%)
Days post transplantation(DPT)
Control
Treatment
F
DAPI NeuroD1 NeuN

Figure S5. scAAV6-NeuroD1 treatment prolongs survival and enhances antitumor immunity in an orthotopic GL261 GBM mouse model.

(A) Schematic of the experimental timeline. C57BL/6 mice were intracranially implanted with 5×10^5 GL261-luc cells on day 0, followed by intratumoral injections of scAAV6-NeuroD1 or PBS (control) on 5-, 10-, and 15-days post transplantation (DPT). Tumor progression was monitored periodically via IVIS (in vivo imaging).

(B) Representative bioluminescence images of mice from control and treatment groups at indicated days post-transplantation (DPT). Control mice showed progressive tumor growth and mortality by day 20, while the treatment group exhibited reduced tumor signals.

(C) Quantitative analysis of total flux (photons/sec) derived from bioluminescence imaging. The treatment group demonstrated a decrease in total flux compared to controls, indicating effective tumor suppression. Data are presented as mean ± SEM. $p = 0.1545$ at 15 DPT by Sidák's multiple comparisons test.

(D) Body weight changes of mice during the study period. The treatment group exhibited a less pronounced decline compared to controls as tumor progressed.

(E) Kaplan–Meier survival curves of control and treated groups (n=6 per group). The treatment group demonstrated a significantly higher survival rate, highlighting the therapeutic benefit of scAAV6-NeuroD1. $p<0.001$ by Log-rank (Mantel-Cox) test.

(F) Representative immunofluorescence image of NeuroD1 (red) and NeuN (green) at 28 days post-tumor transplantation (DPT). The image shows the border region between tumor tissue (right) and normal brain tissue (left), indicated by dense DAPI staining (blue) on the right. NeuroD1 expression (red) is observed along the tumor boundary, with partial co-localization with the neuronal marker NeuN (green), as indicated by yellow arrows. These findings suggest neuronal reprogramming at the tumor–normal interface. Scale bar = 20 μm.

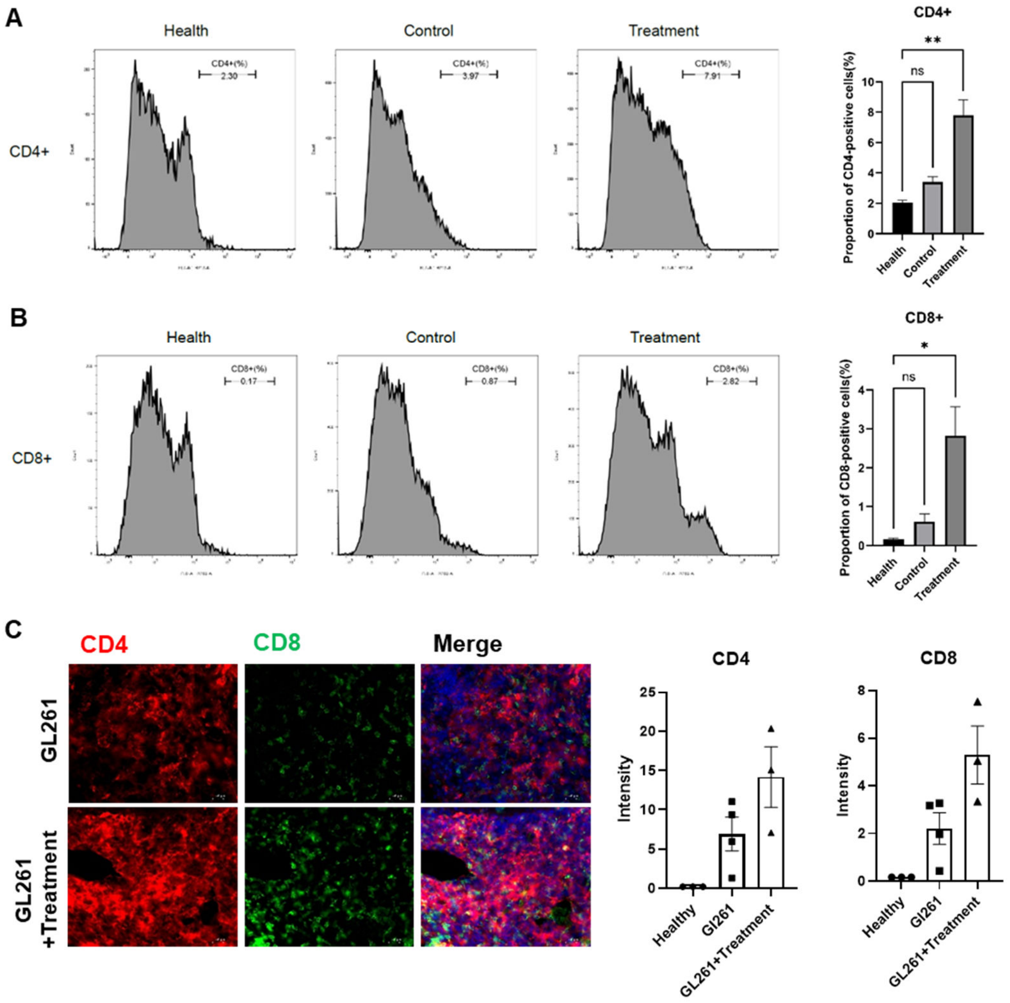

Figure S6. Analysis of T cell infiltration in brain tissues upon scAAV6-NeuroD1 treatment in GL261 GBM mouse model.

(A) Flow cytometry analysis of $CD4^{+}$ T cell infiltration in brain tissues from healthy, tumor-bearing control, and treated mice. Representative flow plots (left) and quantitative summary (right) show the percentage of $CD4^{+}$ T cells among $CD45^{+}$ immune cells.

(B) Flow cytometry analysis of $CD8^{+}$ T cell infiltration under the same conditions. Representative flow plots (left) and quantitative data (right) display the proportion of $CD8^{+}$ T cells.

(C) Immunofluorescence staining of $CD4^{+}$ (red) and $CD8^{+}$ (green) T cells in brain sections, with DAPI (blue) indicating nuclei. Merged images and quantitative fluorescence intensity analysis further confirm T cell recruitment into the tumor region.

Data are presented as mean ± SEM; $*p < 0.05$, $**p < 0.01$ by ordinary one-way ANOVA with Tukey's post hoc test.

Table S1. Clinicopathologic characteristics of patients with high and low expression of NEUROD1 in primary glioma (CGGA cohort: mRNAseq_325)

Table S1			
**Characteristic**	**Low *NEUROD1* expression**	**High *NEUROD1* expression**	**P value**
No. of patients	110[@]	112[@]	
Gender			
Female	39	45	0.4681[a]
Male	71	67	
WHO grade			
II+III	52	85	<0.0001[a]
IV	58	27	
IDH status			
Wild type	74	35[#]	<0.0001[a]
Mutation	36	76[#]	

[@]Grouping followed CGGA survival data.
[#]Patient had no *IDH* status record.
[a]P values were determined by chi-square test.

Table S2. Clinicopathologic characteristics of patients with high and low expression of *NEUROD1* in primary glioma (CGGA cohort: mRNAseq_693)

Table S2			
**Characteristic**	**Low *NEUROD1* expression**	**High *NEUROD1* expression**	**P value**
No. of patients	200[@]	204[@]	
Gender			
Female	81	92	0.3504[a]
Male	119	112	
WHO grade			
II+III	120	151	0.0027[a]
IV	80	53	
IDH status			
Wild type	102[#]	68[#]	<0.0001[a]
Mutation	72[#]	125[#]	

[@]Grouping followed CGGA survival data.
[#]Patients had no *IDH* status records.
[a]P values were determined by chi-square test.

Table S3. Clinicopathologic characteristics of patients with high and low expression of *NEUROD1* in primary glioma (CGGA cohort: mRNA-array_301)

Table S3.			
**Characteristic**	**Low *NEUROD1* expression**	**High *NEUROD1* expression**	**P value**
No. of patients	131@	131@	
Gender			
Female	48	58	0.2081[a]
Male	83	73	
WHO grade			
II+III	66	89	0.0038[a]
IV	65	42	
IDH status			
Wild type	86#	60#	0.0012[a]
Mutation	44#	70#	

@Grouping followed CGGA survival data.
#Patients had no *IDH* status records.
[a]P values were determined by chi-square test.
